# Recent advances in cyclization reactions of *ortho*-substituted *gem*-dibromoolefins

**DOI:** 10.1039/d5ra06293d

**Published:** 2025-10-28

**Authors:** Saideh Rajai-Daryasarei, Navid Ranjbar Naieni, Saeed Balalaie

**Affiliations:** a Department of Chemistry, College of Science, University of Tehran Tehran Iran saeedehrajai@ut.ac.ir; b Peptide Chemistry Research Institute, K. N. Toosi University of Technology P. O. Box 15875-4416 Tehran Iran balalaie@kntu.ac.ir

## Abstract

The preparation of cyclic compounds has received a significant amount of attention, because of their extensive presence in natural products, bioactive molecules, and materials science. Accordingly, the straightforward design and synthesis of cyclic compounds using readily accessible starting materials and reagents are among the main targets of synthetic chemists. In this regard, transformations involving *ortho*-substituted *gem*-dibromoolefins as good building blocks are recognized as powerful and efficient approaches for one-pot synthesis of cyclic compounds. This review focuses on approaches involving *ortho*-substituted *gem*-dibromoolefins that have been developed over the past two decades for the synthesis of cyclic compounds *via* both transition metal-catalyzed and metal-free cyclization methods.

## Abstract 2

The preparation of cyclic compounds has received a significant amount of attention, because of their extensive presence in natural products, bioactive molecules, and materials science.

## Graphical abstract

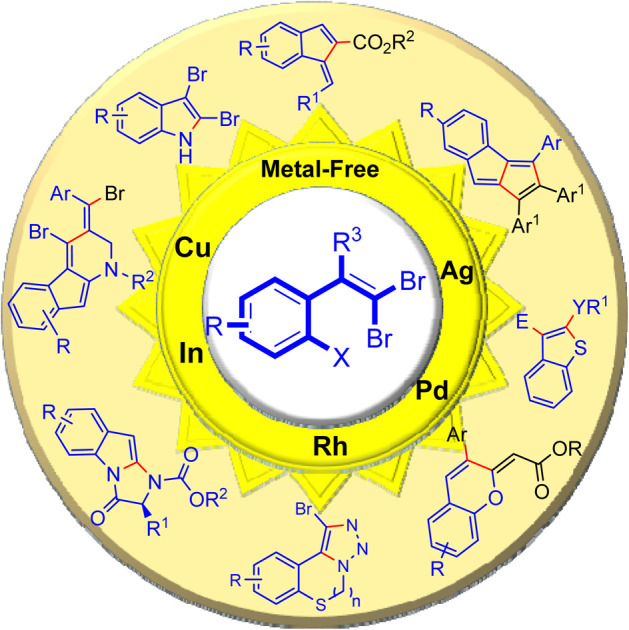

## 1. Introduction

**Scheme 1:**
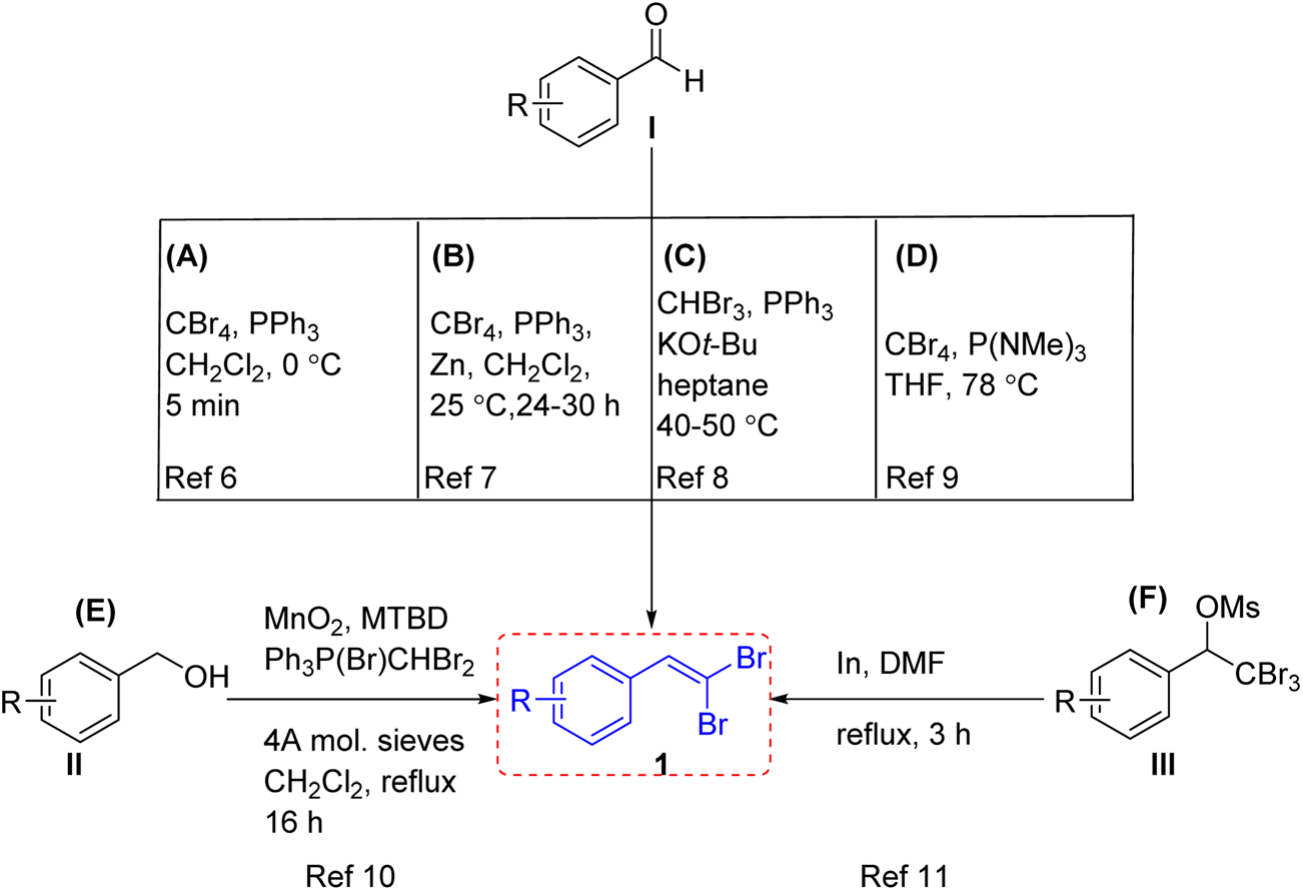
Synthesis of *gem*-dibromovinyl derivatives.

Cyclic organic compounds, including carbo- and heterocycles, are found in a wide range of drugs, biomolecules, natural products, agrochemicals, and biologically active compounds, such as anti-HIV, antidiabetic, antimalarial, antibacterial, antitumor, herbicidal anti-inflammatory, antiallergic, antidepressant, antibiotic, antimicrobial, antiviral, antifungal, anticancer.^1^ Moreover, cyclic compounds have broad applications in the field of materials science, and polymer chemistry, supramolecular chemistry, organic synthesis, and medicinal chemistry.^2^ In light of the importance of this class of organic compounds, significant attention has been paid to the development of efficient and powerful protocols for the synthesis of cyclic structures. Among commonly utilized synthons, *gem*-dibromoolefins have found widespread applications in construction of organic compounds. They can be employed as interesting synthetic intermediates in a variety of non-metal-assisted chemical transformations.^3^ Furthermore, *gem*-dibromoolefins are very prone towards oxidative addition with metal complexes compared to analogous monohaloalkenes, due to the existence of two geminal bromines bonded to the one alkenyl carbon. This makes the vinyl dihalide moiety of *gem*-dibromoolefins an appropriate and versatile bidentate electrophile for organometallic reactions, easily undergoing metal-catalyzed cross-coupling reactions.^4^*Gem*-dibromoolefins can be conveniently prepared by a variety of methods such as Wittig-type reactions, elimination-based reactions, and substitution reactions.^4,5^ For example, in 1962, Ramirez and co-workers using the reaction of aromatic aldehyde with carbontetrabromide and triphenylphosphine, that is converted to (dibromomethylene)triphenylphosphane (Ph_3_P=CBr_2_) *in situ*, in CH_2_Cl_2_ at 0 °C for 5 min can produce *gem*-dibromoolefins 1 (Scheme 1A).^6^ A modified version was later reported by Corey and Fuhs, in which zinc dust was used to reduce the initially produced Br_2_PPh_3_, thereby lowering the required amount of phosphine, improving the yield, and simplifying the separation process (Scheme 1B).^7^ Another approach employed bromoform in place of carbon tetrabromide. The ylide Ph_3_P=CBr_2_, generated from bromoform and PPh_3_ in the presence of KO*t*-Bu, reacted with aromaticaldehyde I to afford *gem*-dibromovinyl derivatives 1, albeit in low yields (Scheme 1C).^8^ A strategy reported by Combret *et al.* uses hexamethylphosphorous triamide [P(NMe_2_)_3_] in place of PPh_3_, affording dibromomethylenation products 1 in satisfactory yields (Scheme 1D).^9^ A method was also developed by Taylor and co-workers, involving a one-pot synthesis of 1,1-dibromoalkenes 1 from primary alcohols II*via* MnO_2_-mediated oxidation followed by a Wittig reaction (Scheme 1E).^10^ An alternative approach utilized indium metal to reduce mesylates of aryl-substituted tribromomethyl carbinols III, providing the corresponding vinylidene dibromides 1 in good yields (Scheme 1F).^11^ In particular, the presence of an *ortho*-substitute to the *gem*-dibromoolefin moiety allows for cyclization reaction, resulting in versatile hetero- and carbocyclic compounds. Generally, the synthesis of *ortho*-substitute to the *gem*-dibromoolefin proceeds in a manner analogous to the approach first reported by Ramirez for the preparation of simpler *gem*-dibromoolefin.

*Ortho*-substituted *gem*-dibromoolefins include a unique combination of steric and electronic features that makes them attractive intermediates for cyclization and addition reactions. The geminal dibromo group is highly reactive and can undergo transformations including metal-catalyzed coupling, nucleophilic addition, and elimination reactions. On the other hand, these compounds have favorable spatial orientation due to the *ortho*-substituent, making them an ideal candidate for intramolecular cyclization, enabling efficient construction of cyclic and polycyclic frameworks. These properties render them valuable in the synthesis of heterocycles and complex molecular scaffolds *via* cascade or stepwise methods. Based on our investigation, no comprehensive report has been found that specifically focuses on the use of *ortho*-substituted *gem*-dibromoolefins in the synthesis of cyclic compounds. This compound contains two reactive sites that facilitate nucleophilic addition reactions. Consequently, we focus on organic reactions that employ these substrates for the construction of cyclic frameworks. This review is organized into two main sections based on the different cyclization reactions of *ortho*-substituted *gem*-dibromoolefins to construct cyclic compounds under transition-metal catalyzed or metal-free conditions. This review encompasses relevant literature published between 2004 and 2025 to highlight the significance of these starting materials in organic synthesis and to illustrate their potential for future applications. The future research for this compound not only affords novel strategies for the synthesis of specific cyclic compounds and complex molecules, but also promotes the development of the transition metal catalyzed reactions. Additionally, substituting precious metals like palladium, rhodium with cheaper and more environmentally friendly metals or organocatalytic systems and metal-free conditions may improve accessibility and reduce environmental impact.^12^

## 2. Transition-metal-catalyzed cyclization reactions

### 2.1. Copper-catalyzed cyclization

Copper catalysts have attracted considerable attention in organic synthesis due to their affordability, abundant availability, and relatively low toxicity.^13^ The versatile nature of copper arises from its ability to readily access multiple oxidation states (Cu^0^, Cu^I^, Cu^II^, and Cu^III^), enabling both one-electron radical pathways and two-electron processes similar to palladium-catalyzed reactions.^14^ Several reviews have highlighted the breadth and significance of copper-catalyzed and copper-mediated reactions.^15^ This section specifically focuses on recent advances in copper-catalyzed cyclizations involving *ortho*-substituted *gem*-dibromoolefins, emphasizing mechanistic insights, substrate scopes, and the formation of valuable heterocyclic frameworks.

**Scheme 2:**
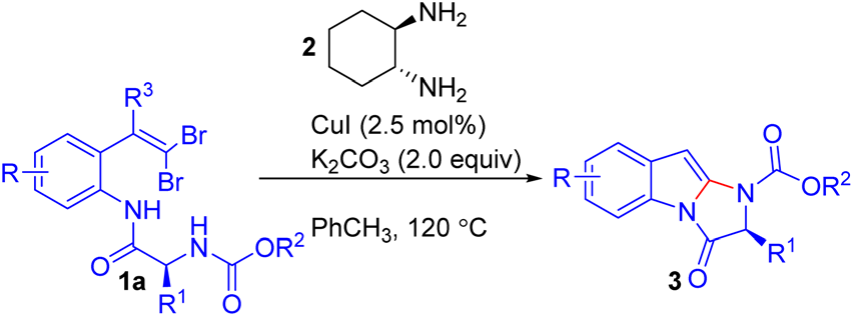
Synthesis of substituted imidazoindolones.

In 2006, Lautens and colleagues reported the first CuI-catalyzed tandem intramolecular amidation of *ortho*-substituted *gem*-dibromoolefins 1a to synthesize substituted imidazoindolones 3 key motifs in bioactive compounds like asperlicin and fumiquinazolines *via* a two-step C–N bond formation sequence involving initial intramolecular amidation and subsequent coupling with a tethered carbamate (Scheme 2).^16^ The authors found that using *trans*-1,2-cyclohexyldiamine 2 with K_2_CO_3_ in toluene at 120 °C gave the best yields and up to 98% enantiomeric excess. The scope of the reaction was explored using a variety of amino acid-derived *ortho*-substituted *gem*-dibromoolefins 1a, showing that alkyl groups, including sterically hindered substituents like isopropyl and isobutyl, were well tolerated. Notably, substrates with heteroatoms or sensitive functional groups, such as carbamates, successfully formed five-membered imidazoindolone rings 3 without competing side reactions. A series of electron-donating and electron-withdrawing groups on the aromatic ring had minimal impact on the efficiency, except for the 5-methoxy group, which slightly reduced the yield due to steric hindrance.

**Scheme 3:**
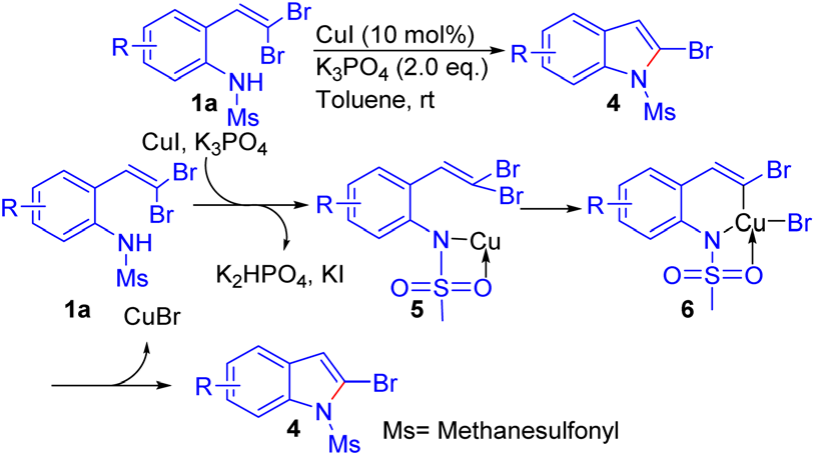
Synthesis of 2-bromoindole derivatives.

Zhang *et al.* described a mild and simple method for synthesizing 2-bromoindoles 4 through a ligand-free CuI-catalyzed intramolecular cross-coupling of *gem*-dibromovinylaniline derivatives 1a (Scheme 3).^17^ The reaction proceeds smoothly in toluene at room temperature using K_3_PO_4_ as the base and CuI as the catalyst, affording the desired 2-bromoindole derivatives 4 in excellent yields. The authors evaluated different *N*-protecting groups on the aniline substrate and identified methanesulfonyl as the most effective, likely due to its coordinating ability and the ease of deprotonation of the aniline's ‘NH’ group by the base. Under these optimized conditions, a variety of 2-bromoindoles 4 were synthesized from *ortho*-substituted *gem*-dibromoolefins 1, including substrates with electron-withdrawing (*e.g.*, 4-Cl, 4-F) and electron-donating (*e.g.*, 4-Me, 4-OMe) substituents. The reaction showed broad functional group tolerance, successfully delivering the desired products 4 even with steric bulk near the dibromoolefin moiety or adjacent to the *N*-Ms group. The proposed mechanism involves initial coordination of CuI to the sulfonamide group, followed by oxidative insertion into the *gem*-dibromoolefin to form a cyclic copper intermediate 6, which subsequently undergoes reductive elimination to furnish the target 2-bromoindoles 4.

**Scheme 4:**
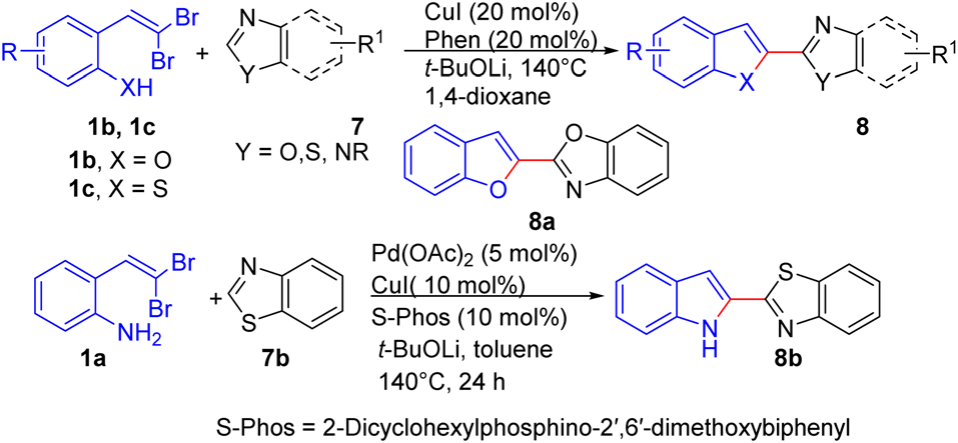
Synthesis of benzofused heteroaryl azoles.

In 2011, Lan and colleagues developed a one-pot Cu(ii)-catalyzed reaction to synthesize benzofused heteroaryl azoles 8 (Scheme 4).^18^ Two bonds including C(sp^2^)–C(sp^2^) and C(sp^2^)–O bonds were constructed in one synthetic operation in the tandem cross-couplings of *ortho*-substituted *gem*-dibromoolefins 1b/1c with azoles 7. It is noteworthy that their strategy is not only appropriate for various benzofused heterocycles 8 (*e.g.*, benzofurans, benzothiophenes, and indole), but also a relatively wide range of azoles 7 including thiazoles, oxazoles, imidazoles, and oxadiazoles, resulting in good to excellent yields of the desired products 8. Remarkably, the methodology effectively produced 2-(benzofuran-2-yl)-benzoxazole 8a, an active ingredient in sunscreen formulations, with a 68% yield. Several synthesized compounds are crucial biologically active alkaloids and display high potency as antagonists at human A2B adenosine receptors. Although the Cu(i)/Phen catalytic system effectively facilitated the coupling of 2-*gem*-dibromovinylphenol/thiols 1b/1c with various azoles 7b, the synthesis of 2-(indole-2-yl)-azoles 8b from 2-*gem*-dibromovinylbenzenamine 1a posed a challenge. However, a catalyst system generated *in situ* from Pd(OAc)_2_/S-Phos/CuI and *t*-BuOLi provided a viable solution, successfully yielding 2-(indol-2-yl)-benzothiazole 8b with a 52% yield.

**Scheme 5:**
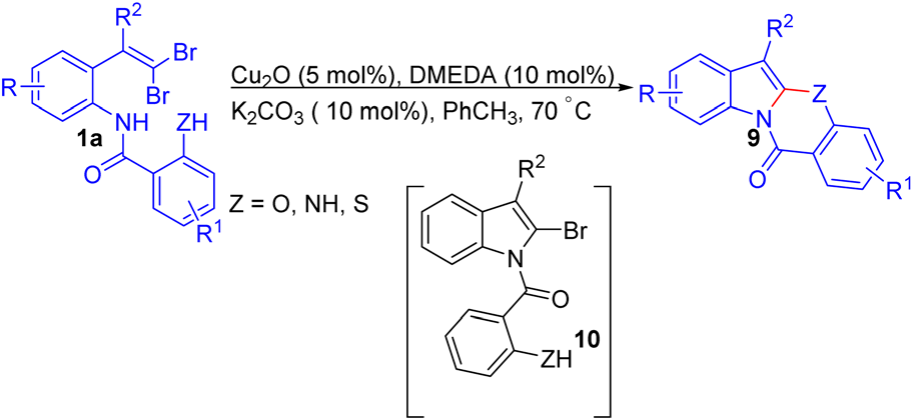
Synthesis of polycyclic indole derivatives.

In 2012, a versatile methodology for synthesizing polycyclic indole derivatives 9 from *gem*-dibromoolefins 1a was introduced by Xia and colleagues (Scheme 5).^19^ This approach utilized a mild and efficient Cu_2_O-catalyzed domino reaction involving intramolecular C–N coupling and C–Z (Z = O, S, N) bond formation. The optimized conditions included Cu_2_O as the catalyst, *N*,*N*′-dimethylethylenediamine (DMEDA) as the ligand, K_2_CO_3_ as the base, all in toluene at 70 °C under N_2_. The method demonstrated broad tolerance, with most substituted *o-gem*-dibromovinyl substrates 1a delivering polycyclic products 9 in excellent yields within 0.5–6 hours. Both electron-donating and electron-withdrawing groups on the indolyl or phenyl rings were tolerated, though substrates with strongly two electron-withdrawing groups (NO_2_) produced only trace amounts of tetracyclic product 9 due to reduced nucleophilicity of both the NH and OH groups. The formation of monocyclized intermediate 10 as the key intermediate in this reaction was suggested by the authors.

**Scheme 6:**
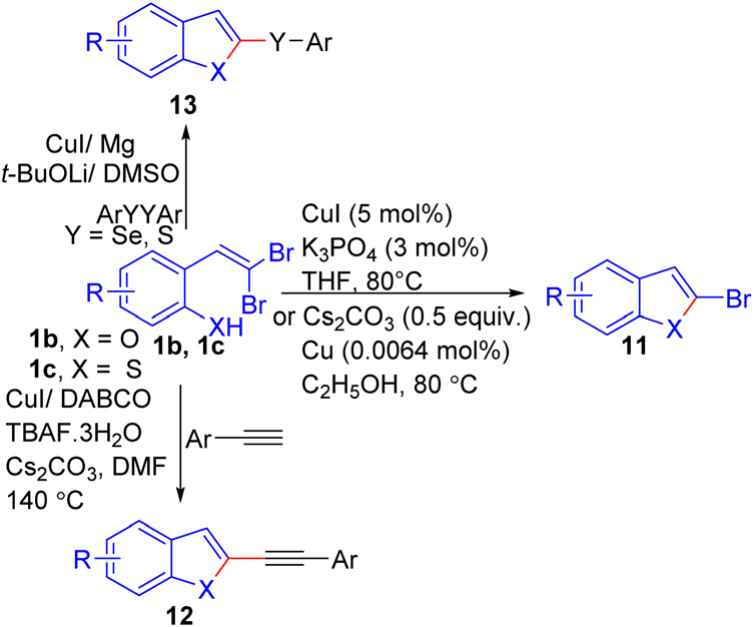
Synthesis of benzofuran/thiophen derivatives.

Several one-pot protocols have also been developed for synthesis of benzofuran/thiophen derivatives 11, 12, 13 using reaction of *ortho*-substituted *gem*-dibromoolefins 1b/1c with other reagents in the presence of Cu catalyst in moderate to excellent yields.^20^ These protocols are summarized in Scheme 6.

**Scheme 7:**
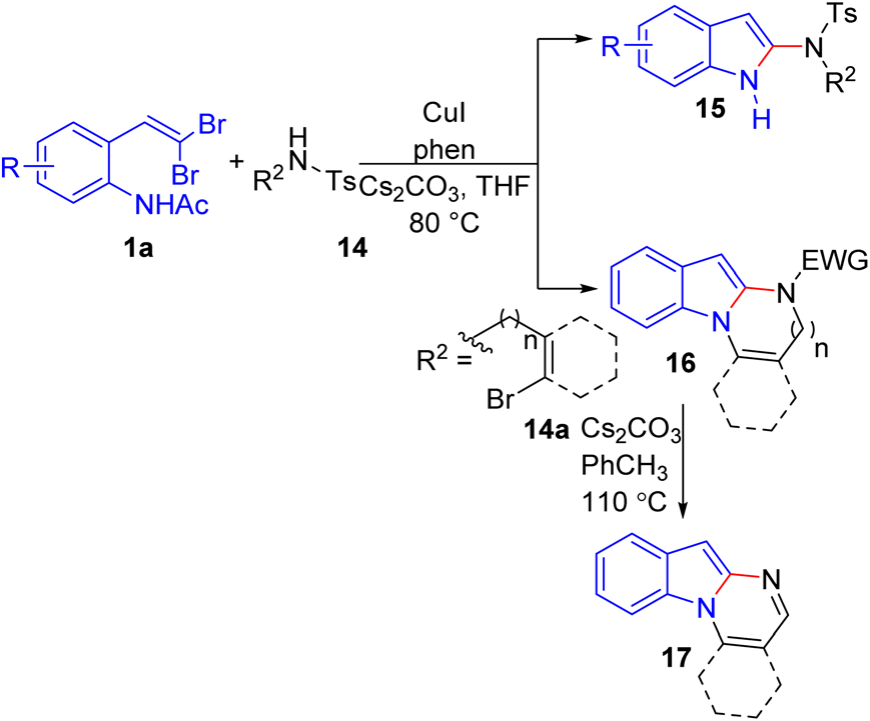
Synthesis of 2-amidoindoles and indolo[1,2-*a*]quinazolines.

In another study, Perumal's group reported a Cu(i)-catalyzed protocol for synthesizing 2-amidoindoles 15 and indolo[1,2-*a*]quinazolines 17 directly from *o-gem*-dibromovinylanilides and sulfonamides 1a in a one-pot process *via in situ* ynamide formation followed by base-promoted intramolecular hydroamidation (Scheme 7).^21^

The optimal conditions involved using 5 mol% of CuI, 10 mol% of 1,10-phenanthroline, and 4 equivalents of Cs_2_CO_3_ in THF at 80 °C. Excellent yields were generally observed for substrates containing electron-rich moieties, halogen substituents, or heterocyclic motifs. Additionally, compatibility with aliphatic substituents such as *n*-butylsulfonamide was demonstrated, affording desired products. Sulfonamides (–Ts or –Ms) were identified as the best coupling partners for the formation of ynamides, resulting in excellent yields of 2-amidoindoles 15 in shorter time durations. The reaction was not successful with acyclic amides or carbamates, which the authors attributed to their lower acidity and the steric hindrance of other nitrogen nucleophiles, making the formation of ynamides very slow and difficult. The reaction was effective with various *N*-tosyl-*o*-bromobenzamides 14a, producing tetrahydroindolo[1,2-*a*]quinazolines 16 in excellent yields. The synthesized tetrahydroindolo[1,2-*a*]quinazolines 16 were then refluxed under basic conditions to yield indolo[1,2-*a*]quinazolines 17.

**Scheme 8:**
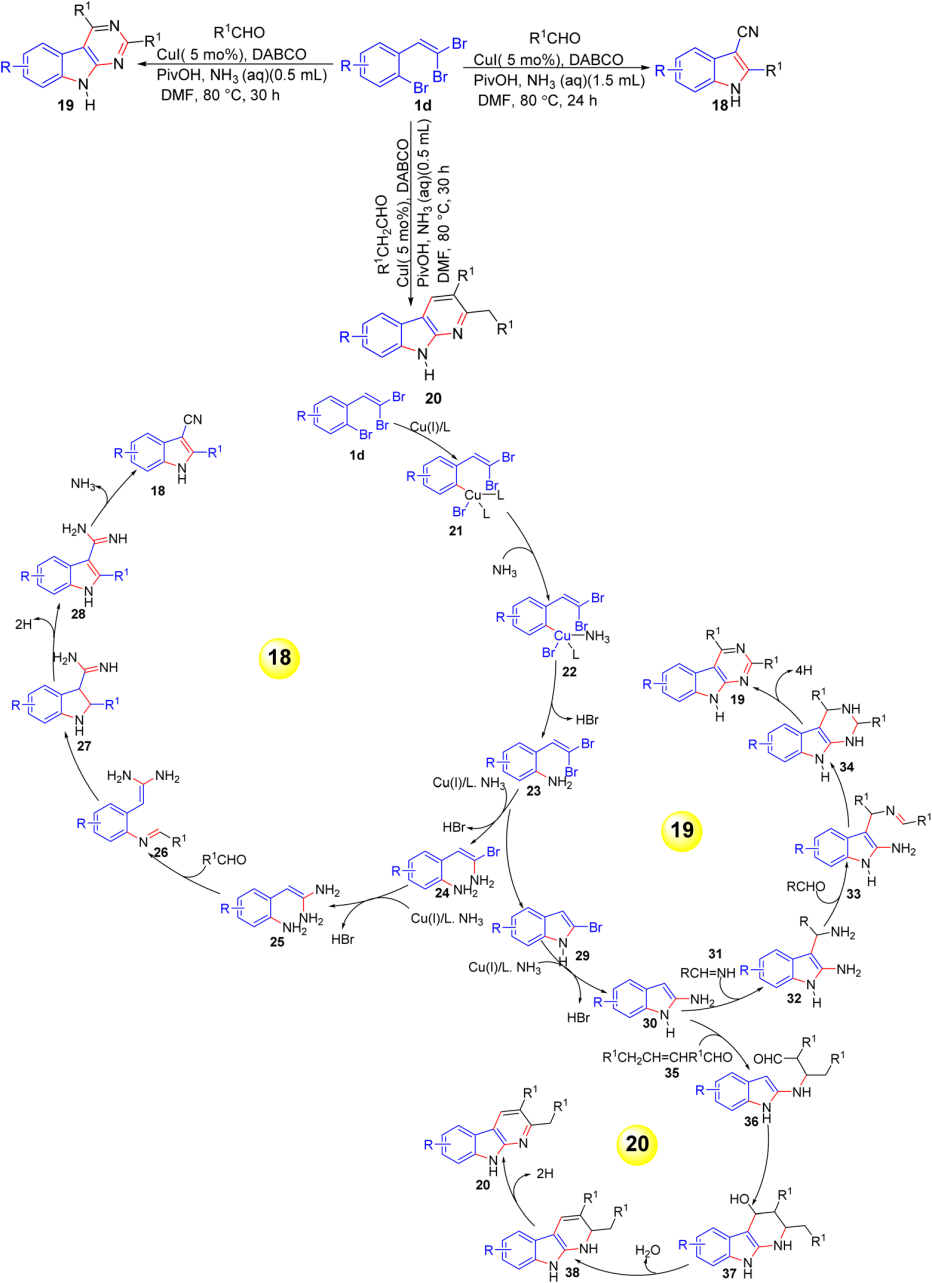
Synthesis of 3-cyano-1*H*-indoles 18, 9*H*-pyrimido[4,5-*b*]indoles 19, and 9*H*-pyrido[2,3-*b*]indoles 20.

In 2015, Li *et al.* developed a copper-catalyzed multicomponent cascade reaction for the synthesis of 3-cyano-1*H*-indoles 18, 9*H*-pyrimido[4,5-*b*]indoles 19, and 9*H*-pyrido[2,3-*b*]indoles 20 from *o-gem*-dibromoolefine derivatives 1d, aldehydes, and aqueous ammonia (Scheme 8).^22^ This one-pot method offers high efficiency and selectivity, with the products determined by controlling the concentration of ammonia and the molar ratio of reagents, furthermore, it tolerates a wide variety of aldehydes, with aryl and alkyl substituents providing good to excellent yields. The authors proposed three distinct mechanisms for the formation of the products during their copper-catalyzed cascade reaction.

#### 2.1.1. Mechanism for 18

The formation of 18 begins with Cu(i)-catalyzed aryl amination of 1-bromo-2-(2,2-dibromovinyl)benzene 1d, producing intermediate 23. Under high ammonia concentration, intermediate 23 undergoes sequential vinyl amination to form intermediates 24 and 25. Condensation of 25 with aldehyde leads to imine 26, followed by intramolecular nucleophilic addition to form intermediate 27. Oxidative dehydrogenation of 27 yields 28, and final ammonia elimination gives the cyanoindole products 18.

#### 2.1.2. Mechanism for 19

Under lower ammonia concentration, the same intermediate 23 first undergoes intramolecular C–N coupling to form 2-bromoindole 29. Subsequent aryl amination results in the formation of 2-aminoindole 30, which then reacts with imine 31 (formed from aldehyde and ammonia) to produce intermediate 32. Further condensation of 32 with another molecule of aldehyde leads to imine 33. Nucleophilic cyclization of 33 affords tetrahydro-1*H*-pyrimido[4,5-*b*]indole 34, which, upon oxidative aromatization, yields 19.

#### 2.1.3. Mechanism for 20

The formation of 20 proceeds with intermediate 30 undergoing Michael addition on an α,β-unsaturated aldehyde 35 formed from self-aldol condensation of acetaldehyde, leading to access intermediate 36. The resulting adduct 36 undergoes intramolecular aldol-type cyclization to form intermediate 37 and 38. Oxidative dehydrogenation of 38 gives the final pyrido[2,3-*b*]indole product 20.

**Scheme 9:**
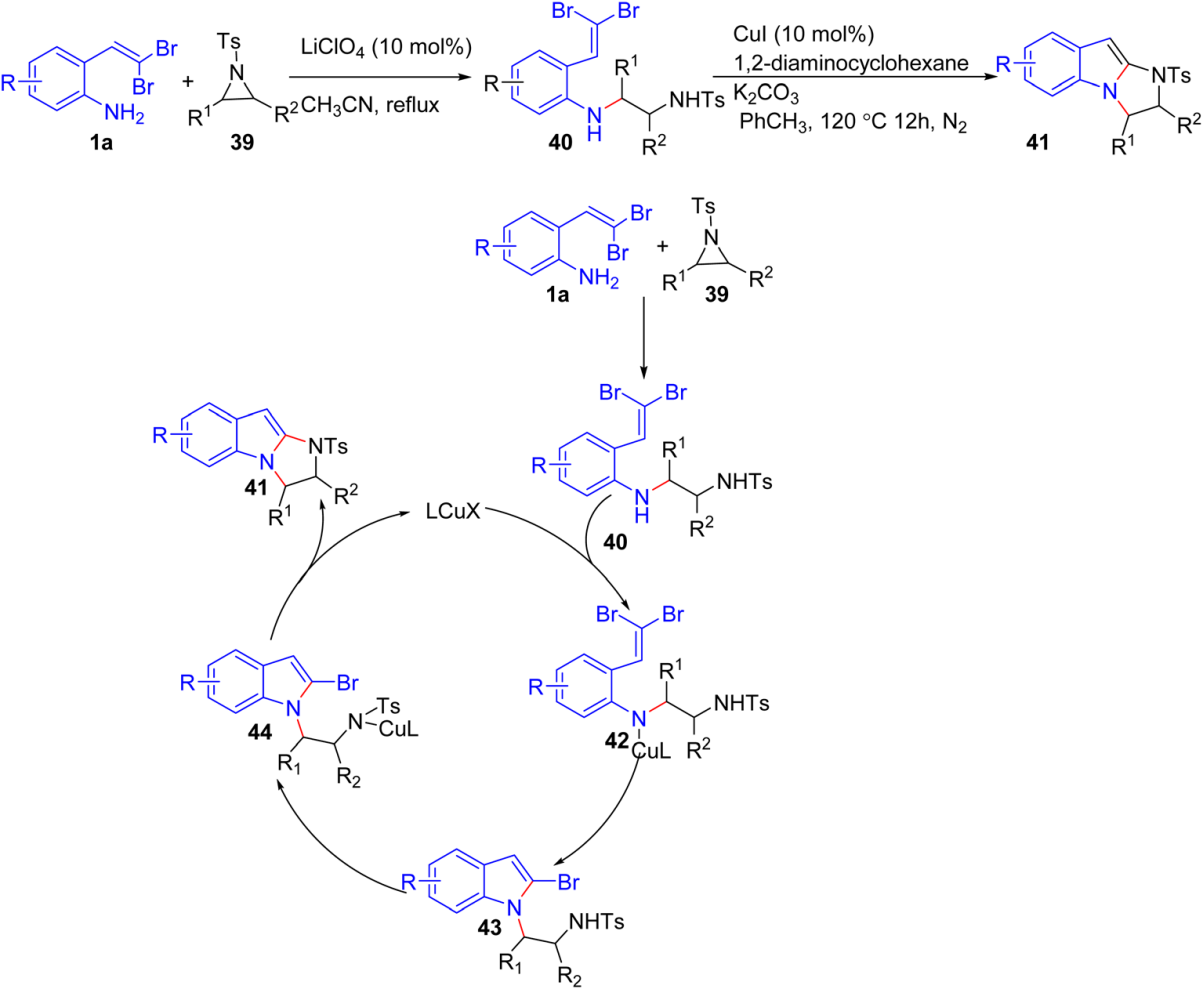
Synthesis of substituted imidazoindoles.

Ghorai *et al.* developed a new synthetic route for producing substituted imidazoindoles 41 in high yields with excellent ee values (Scheme 9).^23^ Activated aziridines 39 undergo ring-opening reactions with *o-gem*-dibromovinylanilines 1a in the presence of LiClO_4_ as a Lewis acid, producing compounds 40, then, this reaction is followed by a copper-catalyzed domino strategy two consequent C–N coupling to formation of imidazoindoles 41. Under optimized CuI-catalyzed conditions, compounds 40 formed cyclized products 41 with good to excellent yields. It is worth that, 2-bromobenzoheterocycle 43 is as key intermediate in this transformation.

**Scheme 10:**
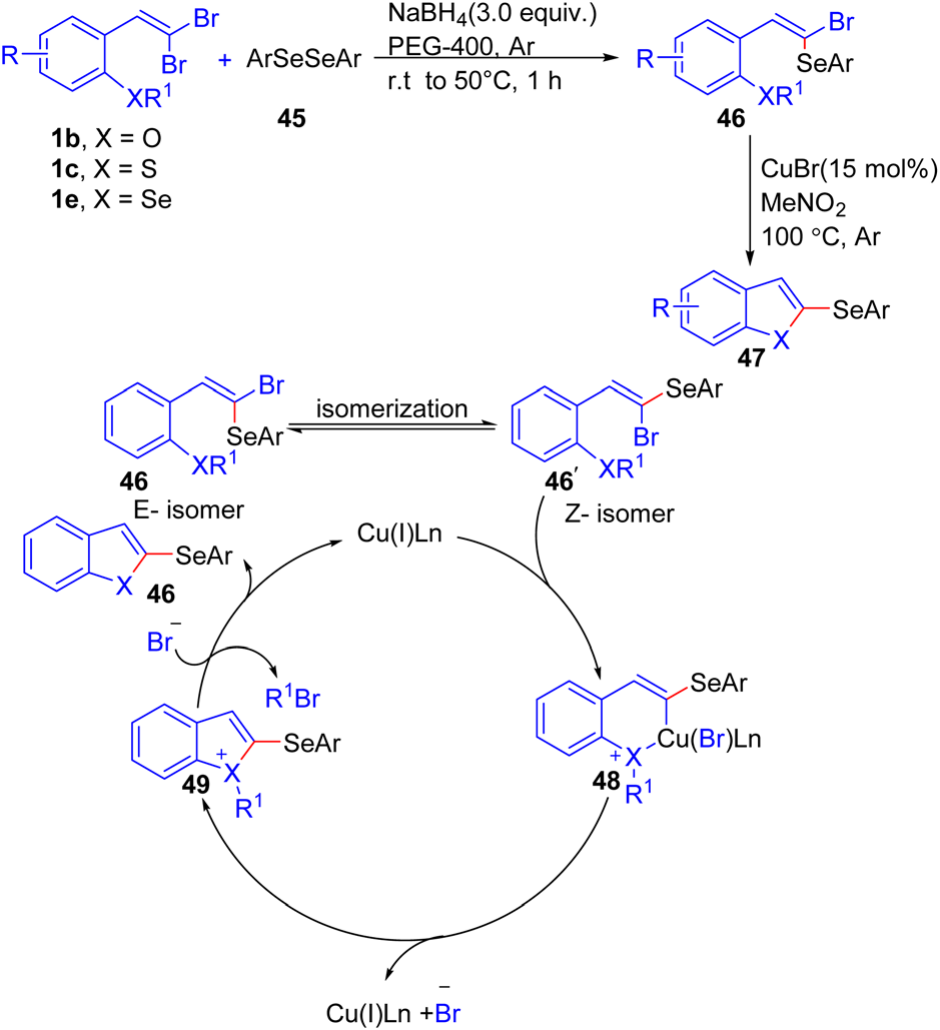
Synthesis of 2-(arylselanyl)benzo[*b*]chalcogenophenes.

A novel method to access 2-(arylselanyl)benzo[*b*]chalcogenophenes 47 through Cu(i)-catalyzed annulation of vinyl selenides 46 that is formed from reaction of *gem*-dibromoolefins 1b/1c/1e with diaryl diselenides 45, has been documented by Perin *et al.* (Scheme 10).^24^ They were the first group to report a general protocol to access seleno-functionalized benzo[*b*]chalcogenophenes 47. First, by using NaBH_4_ as a reducing agent and PEG-400 as the solvent under inert conditions, *ortho*-substituted *gem*-dibromoolefins 1 were reacted with diaryl diselenides 45, affording (*E*)-1-bromo-1-arylselenoalkenes 46 as the products. Then, by reacting 46 with CuBr in nitromethane, 2-(arylselanyl)benzo[*b*]chalcogenophenes 47 were obtained with good yields. The authors proposed this process begins with an isomerization step, converting the *E*-isomer 46 to the *Z*-isomer 46′, which is better suited for oxidative addition to the Cu(i) species, forming the selenonium intermediate 48. Remarkably, experimental evidence supports this hypothesis, as a control experiment using the pure *E*-isomer 46 under the reaction conditions resulted in 45% isomerization to the *Z*-isomer 46′. Subsequently, an Ullmann-type coupling generates intermediate 48, releasing the copper catalyst and bromide. Finally, the bromide acts as a nucleophile, attacking the alkyl group *via* an S_N_2 mechanism to produce the desired product 47 and 1-bromoalkane as a by-product (Scheme 10).

**Scheme 11:**
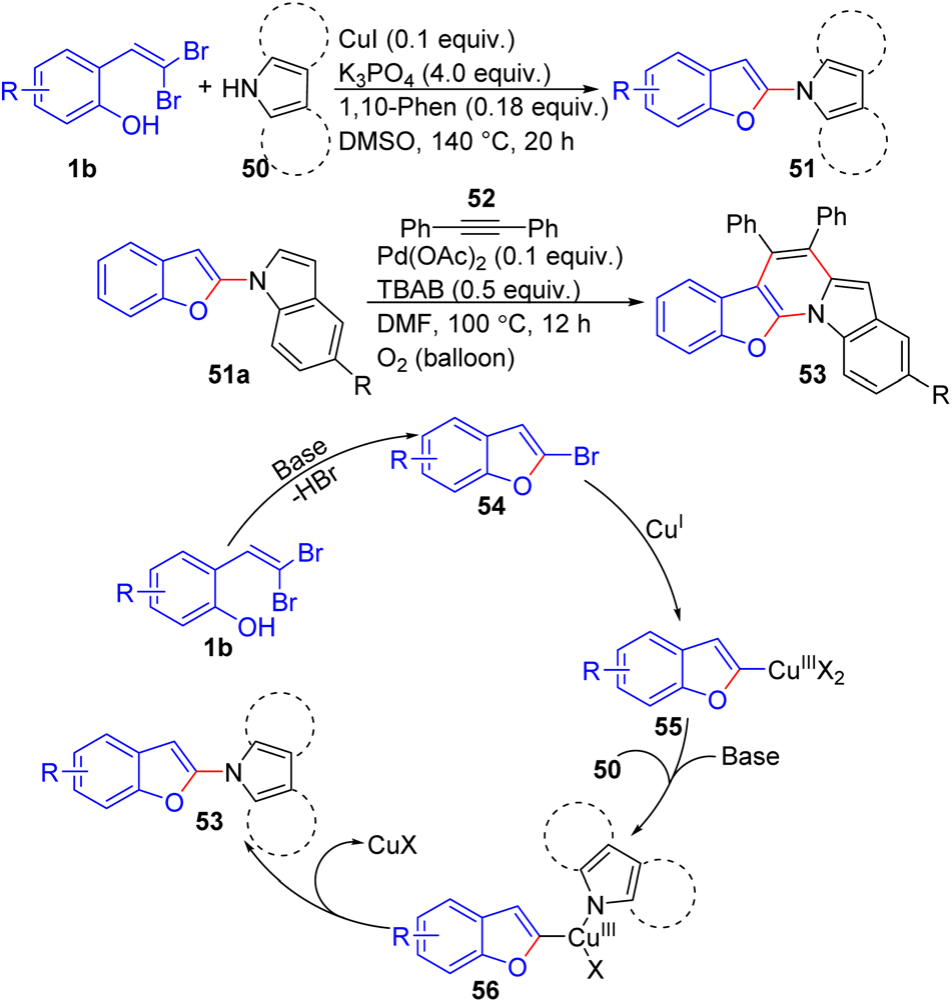
Synthesis of 1-(2-benzofuryl)-*N*-heteroarenes.

Under copper-catalyzed tandem reaction conditions, Rao and Islam developed a versatile method for the synthesis of 1-(2-benzofuryl)-*N*-heteroarenes 51 from *ortho*-substituted *gem*-dibromoolefins 1b, demonstrating a broad substrate scope and high yields (Scheme 11).^25^ Further, these products were effectively utilized in the formation of polycyclic benzofuro-indolo-pyridine architectures 53 through palladium-catalyzed dehydrogenative coupling. The optimized reaction conditions for the copper-catalyzed cross-coupling of 2-(2,2-dibromovinyl)phenol 1b and indole 50 were found to be DMSO as the solvent, K_3_PO_4_, CuI, and 1,10-phenanthroline as the base, copper catalyst, and additive, respectively, at 140 °C for 20 hours. The reported methodology for copper-catalyzed cross-coupling reactions of *o-gem*-dibromides 1b with *N*-heteroarenes 50 proved to be versatile and efficient, yielding a range of bis-heterocycles 51 in good to excellent yields regardless of the presence of electron-donating or -withdrawing functional groups. The mechanism the authors proposed, involves a base-promoted cyclization of 2-(2,2-dibromovinyl)phenol 1b to form 2-bromobenzofuran 54. Then, intermediate 54 is converted to complex 55 in the presence of Cu(i), which, complex 55 reacts with the *N*-heteroarene 50 to generate the highly reactive Cu(iii) complex 56. Finally, reductive elimination reaction of intermediate 56 delivers desired the 1-(2-benzofuryl)-*N*-heteroarenes 53.

### 2.2. Palladium-catalyzed cyclization

Palladium is widely regarded as one of the most versatile and commonly employed transition metals in heterocyclic synthesis, owing to its ability to mediate diverse reactions under mild conditions. Its broad utility originates from the accessible interconversion among its oxidation states (Pd^0^, Pd^II^, and Pd^IV^), each exhibiting distinct chemical behaviors. Palladium catalysis typically requires only catalytic quantities of metal and exhibits exceptional functional group tolerance, usually minimizing the need for protecting groups. This section highlights recent developments in palladium-catalyzed transformations of *ortho*-substituted *gem*-dibromoolefins.^26^

**Scheme 12:**
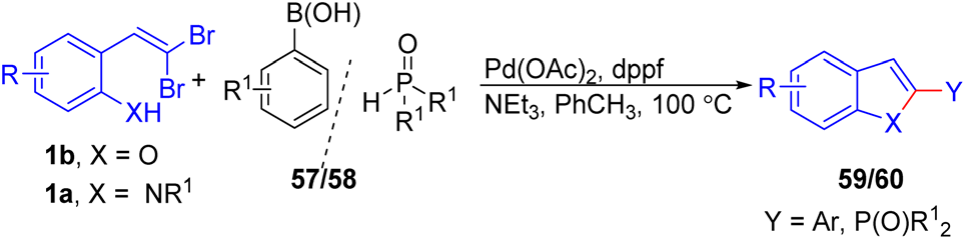
Synthesis of 2-functionalized benzo[*b*]furans and indoles.

Thielges *et al.* developed a tandem Pd-assisted cyclization–coupling reaction for the synthesis of 2-functionalized benzo[*b*]furans and indoles 59/60 starting from *ortho*-substituted *gem*-dibromoolefins 1b, 1a and boronic acids 57/dialkylphosphites 58 (Scheme 12).^27^ By employing palladium acetate [Pd(OAc)_2_] and 1,1′-bis(diphenylphosphino)ferrocene (dppf) as a ligand in toluene with triethylamine as base, the reaction achieves excellent yields while minimizing side products.

**Scheme 13:**
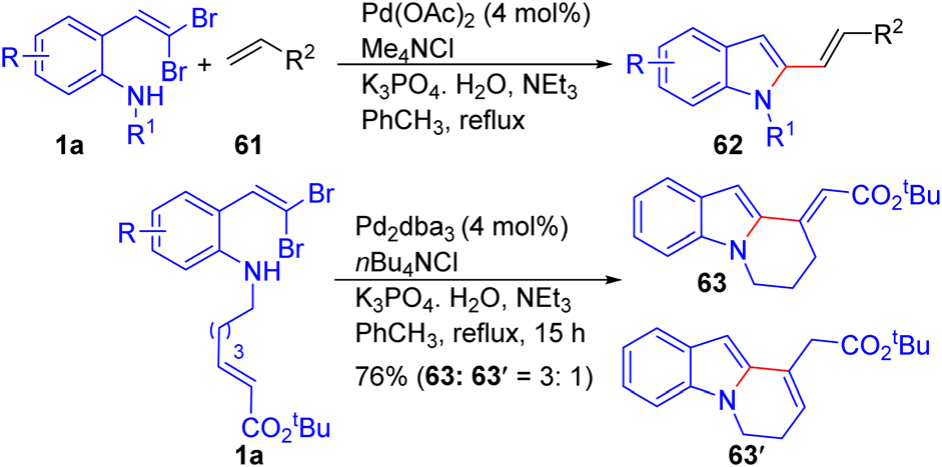
Synthesis of 2-vinylic indoles and their tricyclic derivatives.

Another great example of utilizing *o-gem*-dibromovinylanilines 1a to construct complex indole systems is provided by Lautens *et al.*, who reported a Pd-catalyzed tandem C–N/Heck reaction for the synthesis of 2-vinylic indoles 62 and their tricyclic derivatives 63/63′ in the presence of Me_4_NCl instead of expensive phosphine ligands (Scheme 13).^28^ The authors demonstrated that the reaction tolerates a broad range of alkenes 61, including those bearing electron-donating (*e.g.*, methoxy) and electron-withdrawing groups (*e.g.*, nitrile), as well as functional groups like esters and sulfones, showcasing the versatility of this method. Additionally, a variety of *N*-substituted dibromovinylanilines 1a were compatible, providing 2-vinylic indoles 62 with diverse substitution patterns. The enoate 1 was employed in the presence of tris(dibenzylideneacetone)dipalladium (Pd_2_dba_3_) (4 mol%) with *n*-Bu_4_NCl (1 equiv.) and NEt_3_/K_3_PO_4_·H_2_O in toluene at 120 °C, for this study, and gave the desired tricyclic adduct 63 in good yield.

**Scheme 14:**
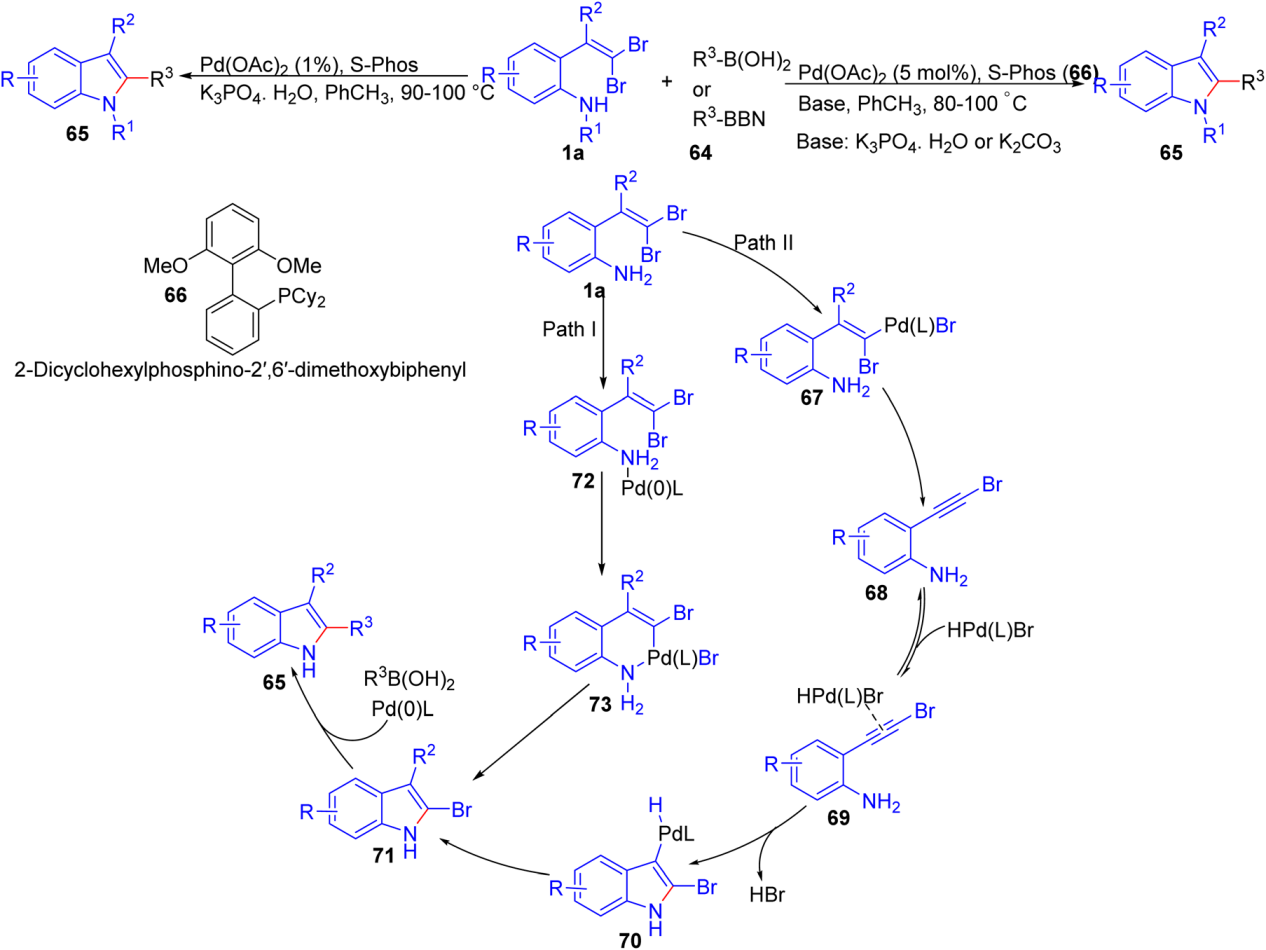
Pd-catalyzed tandem reaction to formation functionalized indoles.

In 2005, Fang and Lautens reported a protocol to generate 2-substituted indoles 65*via* a Pd-catalyzed tandem C–N/Suzuki–Miyaura coupling starting from dibromovinylanilines (Scheme 14).^29^ Optimized reaction conditions were Pd(OAc)_2_ coupled with Buchwald's S-Phos ligand 66 in the presence of K_3_PO_4_/H_2_O in toluene at 90–100 °C. Three years later, they further developed the same methodology, and proposed a possible mechanism and expanded the scope (Scheme 14).^30^ The reaction scope was explored with various *gem*-dihalovinylaniline substrates 1a, demonstrating broad functional group tolerance across both electron-rich and electron-deficient substituents. A range of aryl, alkenyl, and alkyl boron reagents 64 were successfully incorporated, giving the desired indole products 65 in good to excellent yields. The versatility of this methodology is further highlighted by its ability to synthesize 2,3-disubstituted and 1,2,3-trisubstituted indoles 65 through a sequential coupling strategy. Based on several control experiments, the authors proposed two possible pathways: one involves initial alkynyl 68 formation (path II) followed by 5-*endo-dig* cyclization to generate the C–N bond before undergoing Suzuki coupling, while the alternative is a direct Buchwald–Hartwig amination (path I) followed by the Suzuki coupling.

**Scheme 15:**
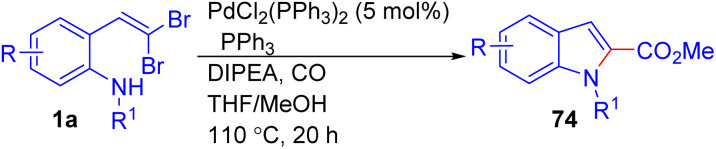
Synthesis of 2-carboxyindoles.

Alper and their group reported a tandem Pd-catalyzed N, C-coupling/carbonylation sequence for the synthesis of 2-carboxyindoles 74 from *gem*-dibromovinyl aniline substrates 1a (Scheme 15).^31^ This reaction employs PdCl_2_(PPh_3_)_2_ in a mixture of THF and methanol under 10 atm of CO at 110 °C, affording the target 2-carboxyindole derivatives 74 in good to excellent yields. The authors noted that the combination of THF and methanol as solvents significantly enhanced product formation compared to using either solvent alone. The protocol showed extensive functional group tolerance, accommodating halogen substituents (*e.g.*, chlorine, fluorine), as well as electron-donating (*e.g.*, methoxy, methyl), without significantly affecting the reaction efficiency.

**Scheme 16:**
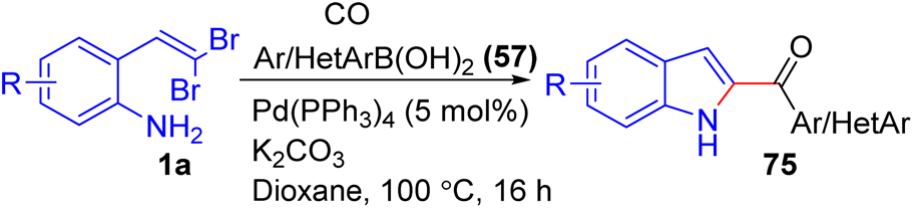
Synthesis of 2-aroyl- and 2-heteroaroylindoles.

Florent *et al.* disclosed a palladium-catalyzed one-pot domino reaction for synthesizing 2-aroyl- and 2-heteroaroylindoles 75 from *o-gem*-dibromovinylanilines 1a, boronic acids 57, and carbon monoxide (Scheme 16).^32^ The reaction, using Pd(PPh_3_)_4_ and K_2_CO_3_ in dioxane under 12 bar of CO at 100 °C, proceeds *via* C, N-coupling, carbonylation, and Suzuki coupling, yielding 2-aroylindoles 75 in moderate to good yields. The scope of this palladium-catalyzed domino reaction demonstrated notable versatility, accommodating a wide range of *gem*-dibromovinylanilines 1a and boronic acid derivatives 57. Electron-rich boronic acids 57, such as 4-methoxyphenyl and 3,4,5-trimethoxyphenyl, gave higher yields, while sterically hindered substrates like 2-methoxyphenylboronic acid resulted in slightly lower efficiency. Electron-deficient and halogen boronic acids 57, such as 4-chlorophenyl and 4-trifluoromethylphenyl, were also compatible, producing the desired products 75 in good yields. Heteroaryl boronic acids 57, including thiophene, benzofuran, and dibenzofuran derivatives, were successfully incorporated, broadening the method's applicability to heterocyclic frameworks. However, reactions involving isoquinolin-3-boronic acid were less efficient, which the authors attributed to interference from the nitrogen atom on the boronic acid 57, resulting in lower yields of the desired products 75. The reaction also tolerated various substituents on the *o-gem*-dibromovinylaniline backbones 1a, with electron-donating groups enhancing yields while electron-withdrawing groups showed moderate reactivity.

**Scheme 17:**
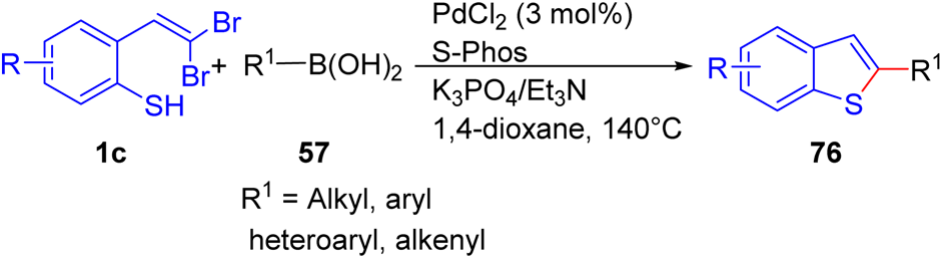
Synthesis of benzothiophenes.

In 2009, Lautens and their colleagues described the first example of a tandem catalytic process that incorporates C–S coupling, and the first example of the palladium-catalyzed reaction of a *o-gem*-dibromovinylthiol 1c to access benzothiophenes 76 in moderate to good yields (Scheme 17).^33^ Utilizing PdCl_2_/S-Phos as their catalytic system, they synthesized a series of functionalized benzothiophenes 76*via* C–S bond-forming and Suzuki–Miyaura coupling reaction. Electron-poor, sterically hindered boronic acids and heteroaromatic boronic acids 57 displayed reactivity comparable to electron-rich 3,4- dimethoxyphenylboronic acid, yielding desired products 76 up to 99%. Both aryl and vinyl trifluoroborate salts were successfully coupled, albeit requiring an increase in the amount of base used. Remarkably, they investigated trialkyl boranes and boronic esters as a patterner that provided access to corresponding products with a good yield.

**Scheme 18:**
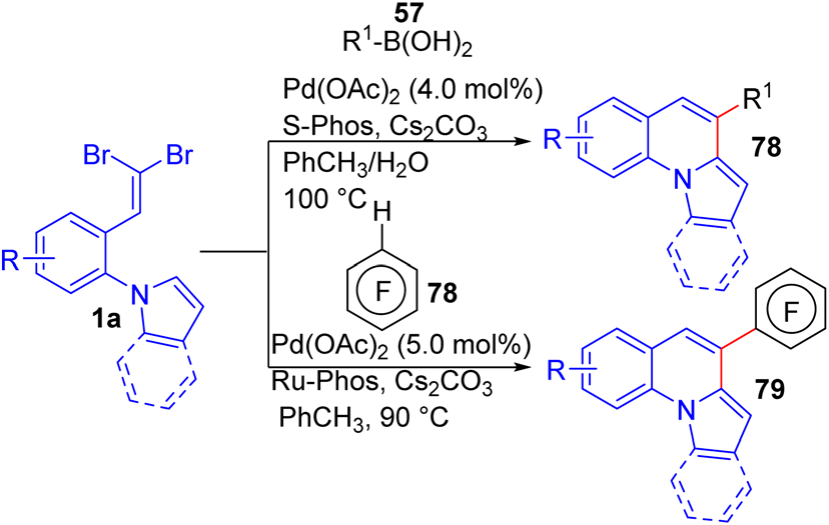
Synthesis of pyrrolo[1,2-*a*]quinoline derivatives.

Chai and Lautens disclosed a highly efficient, water-accelerated, palladium-catalyzed reaction of *ortho*-substituted *gem*-dibromoolefins 1a with a boronic acid derivatives 57*via* a tandem Suzuki–Miyaura coupling and direct arylation process in the presence of S-Phos as ligand and Cs_2_CO_3_ as base in toluene at 100 °C to access pyrrolo[1,2-*a*]quinolines 78 (Scheme 18).^34^ The scope of the reaction was explored using diverse electron-rich and electron-poor boronic acids 54 and substituents on the aromatic ring, revealing excellent compatibility. However, very electron-poor or -rich boronic acids 57 showed reduced yields. The methodology was extended to alkenyl, alkyl, and sterically hindered boronic acids, achieving good results. Substituents on the dibromo alkene partner 1a were varied, with moderate to excellent yields obtained. Heteroaromatic ring systems like indole and pyridine derivatives successfully led to formation products. It is worth mentioning that, the water has a dramatic effect on reactivity of the substrate and the decreasing of the byproduct. Three years later, Wu *et al.* also reported the synthesis of 4-polyfluoroaryl pyrrolo[1,2-*a*]quinolines 79*via* a palladium-catalyzed C–H bond activation reaction of *ortho*-substituted *gem*-dibromoolefins 1a bearing pyrrole moieties with polyfluoroarenes 78 under similar reaction conditions. By this methodology, a study on the reaction scope using several electron-rich and electron-deficient substrates was performed, allowing the synthesis of desired products 79 in poor to very good yields.^35^

**Scheme 19:**
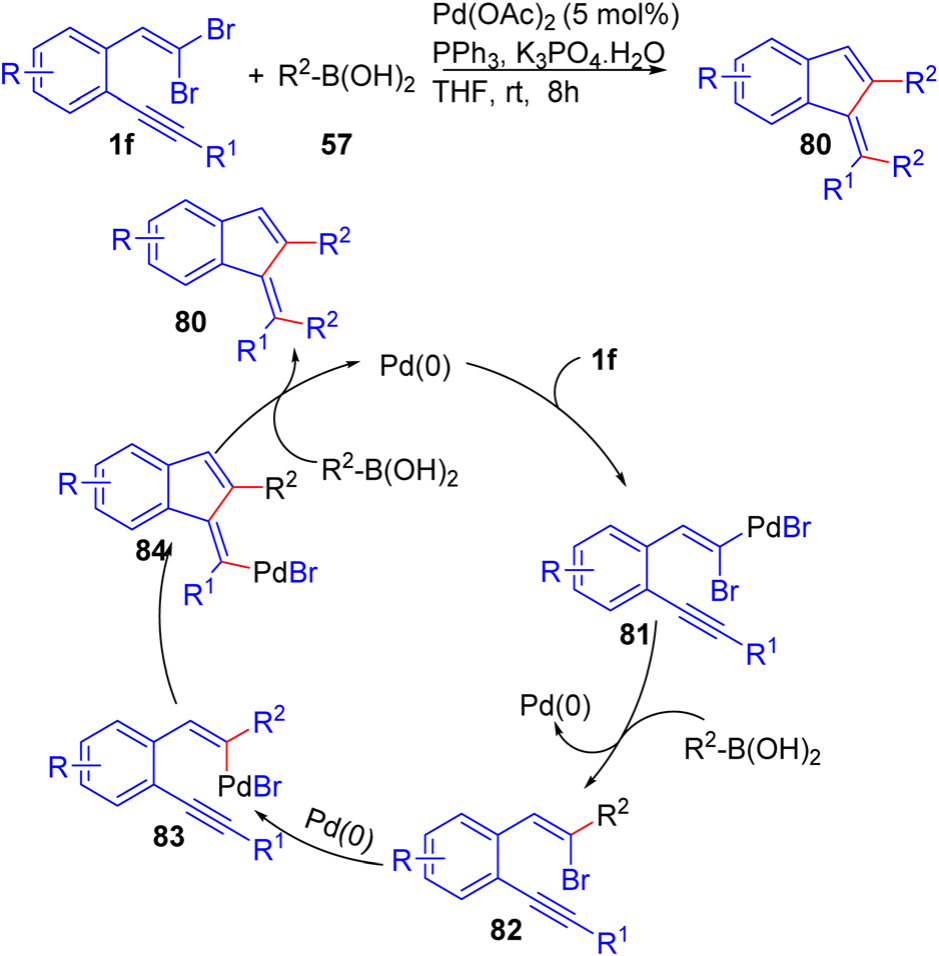
Synthesis of 1-methyleneindenes.

Wu's group developed a method to synthesize 1-methyleneindenes 80 using *ortho*-substituted *gem*-dibromoolefins 1f in a palladium-catalyzed tandem reaction with arylboronic acids 57, efficiently yielding functionalized 1-methyleneindenes 80 under mild conditions with high selectivity (Scheme 19).^36^ Mechanistically, the reaction follows a sequence of palladium-catalyzed oxidative additions, transmetallation, and reductive eliminations, where the *ortho*-substituted *gem*-dibromoolefins 1f undergoes key transformations, allowing for subsequent coupling and cyclization steps. The reaction scope demonstrated broad versatility, accommodating a variety of arylboronic acids 57 with both electron-donating and electron-withdrawing substituents. Even alkyl-substituted boronic acids, such as *n*-butylboronic acid, participated smoothly, further highlighting the flexibility of the method. The reaction also tolerated steric hindrance around the aryl group, with *o*-substituted arylboronic acids showing good reactivity. Additionally, the use of 1-(2,2-dibromovinyl)-2-alkynylbenzenes 1f proved efficient in delivering the desired products 80, with the reaction proceeding smoothly under mild conditions.

**Scheme 20:**
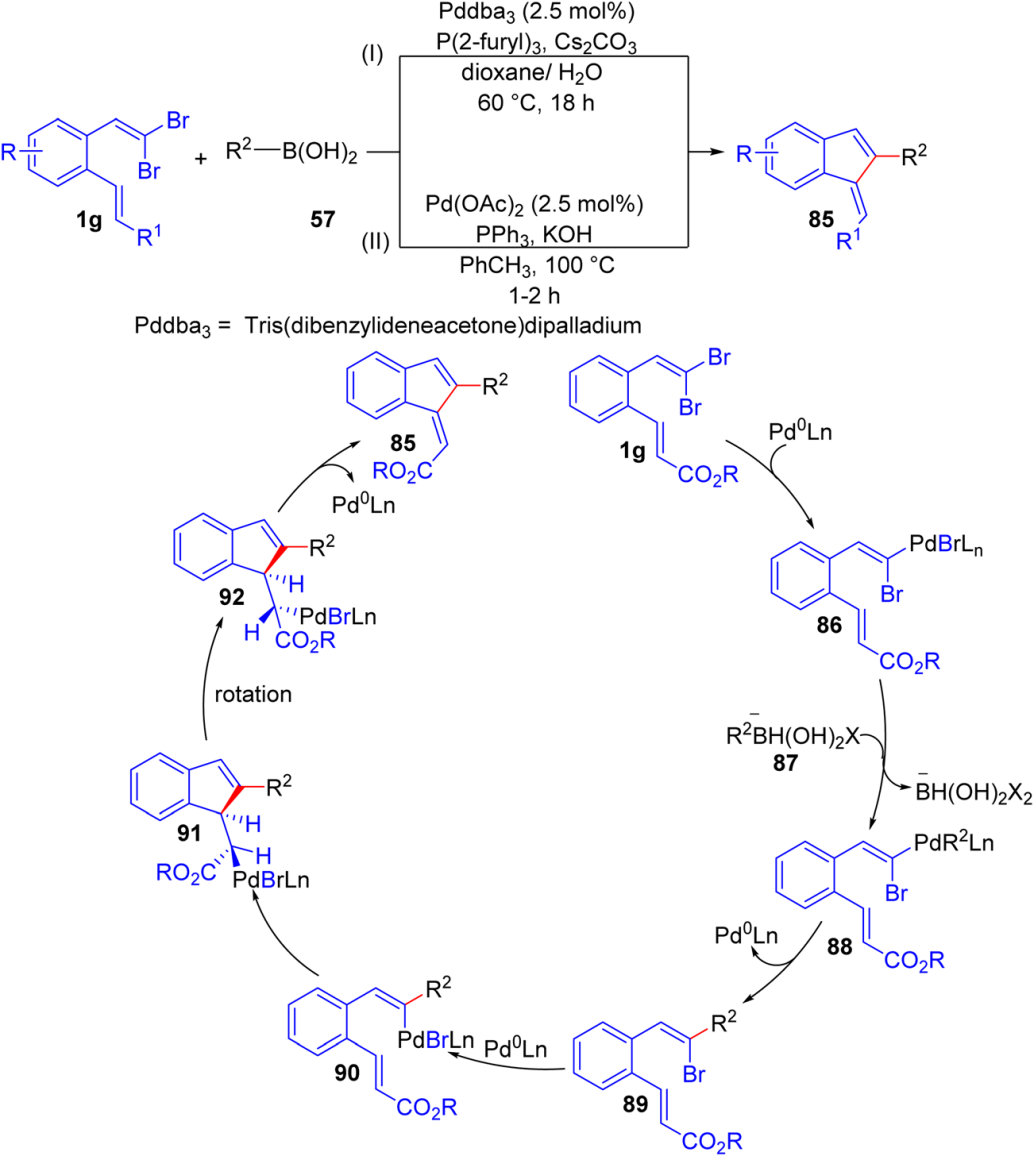
Pd-catalyzed tandem Suzuki/intramolecular Heck reaction of *o*-substituted *gem*-dibromoolefins and boronic acid.

Bryan and Lautens successfully developed a Pd-catalyzed tandem Suzuki/intramolecular Heck reaction of *o*-substituted *gem*-dibromoolefins 1g and boronic acid 57 to access methylenindene scaffolds 85, employing Pd_2_dba_3_ as catalysts, and Cs_2_CO_3_ as the base (Scheme 20(I)).^37^ The choice of ligand proved crucial in selectivity of the reaction, and trifurylphosphine (TFP) was selected as the best ligand in this transformation. The reaction accommodated diverse boronic acids 57 and Heck acceptors, with improved efficiency for electron-rich systems. Electron-poor substrates and steric hindrance reduced yields and reaction rates, respectively. A thiophenyl substrate 1g showed slow reactivity due to sulfur coordination, hindering the Heck process and resulting in low yields. Mechanistic pathway involves oxidative addition of *o*-substituted *gem*-dibromoolefins 1g to Pd^0^, followed by transmetalation with a boronate 87, and reductive elimination of intermediate 89, regenerating the Pd^0^ species. Then, intermediate 89 is converted to the alkenylpalladium 90 in the presence of Pd^0^. Finally, carbopalladation of 90 followed by β-hydride elimination affords the methyleneindene 85. In the same year, Wu *et al.* published a synthesis of 1-methylene-1*H*-indenes 85*via* same starting materials in the presence of Pd(OAc)_2_ (2.5 mol%) and PPh_3_ (5 mol%) as catalyst with KOH (3.0 equiv.) as base in toluene (Scheme 20(II)).^38^ Short reaction time is the most important feature of this method compared to the reported method by Lautens.

**Scheme 21:**
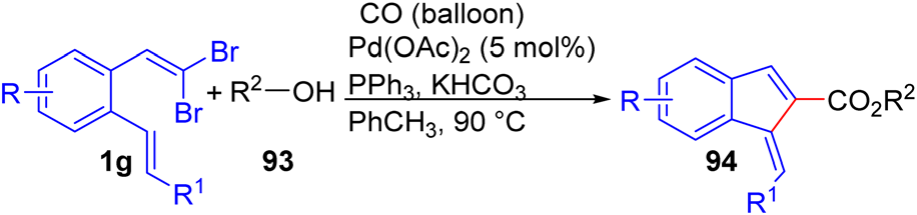
Synthesis of 1-methylene-1*H*-indene-2-carboxylates.

Ye and Wu described a palladium-catalyzed cyclization of *o*-substituted *gem*-dibromoolefins 1g, and carbon monoxide, with phenol or alcohol 93 to produce 1-methylene-1*H*-indene-2-carboxylates 94 through a cascade process that integrated carbonylation and a Heck reaction (Scheme 21).^39^ They believed that acetate anion in the palladium acetate is necessary for this transformation, because, other palladium source led to suppress formation of desired product 94. The presence of various functional groups on the aromatic ring of *o*-substituted *gem*-dibromoolefins 1g generally resulted in good products 94 yield. However, replacing the R^1^ position with a ketone or aryl group led to only trace amounts of product formation.

**Scheme 22:**
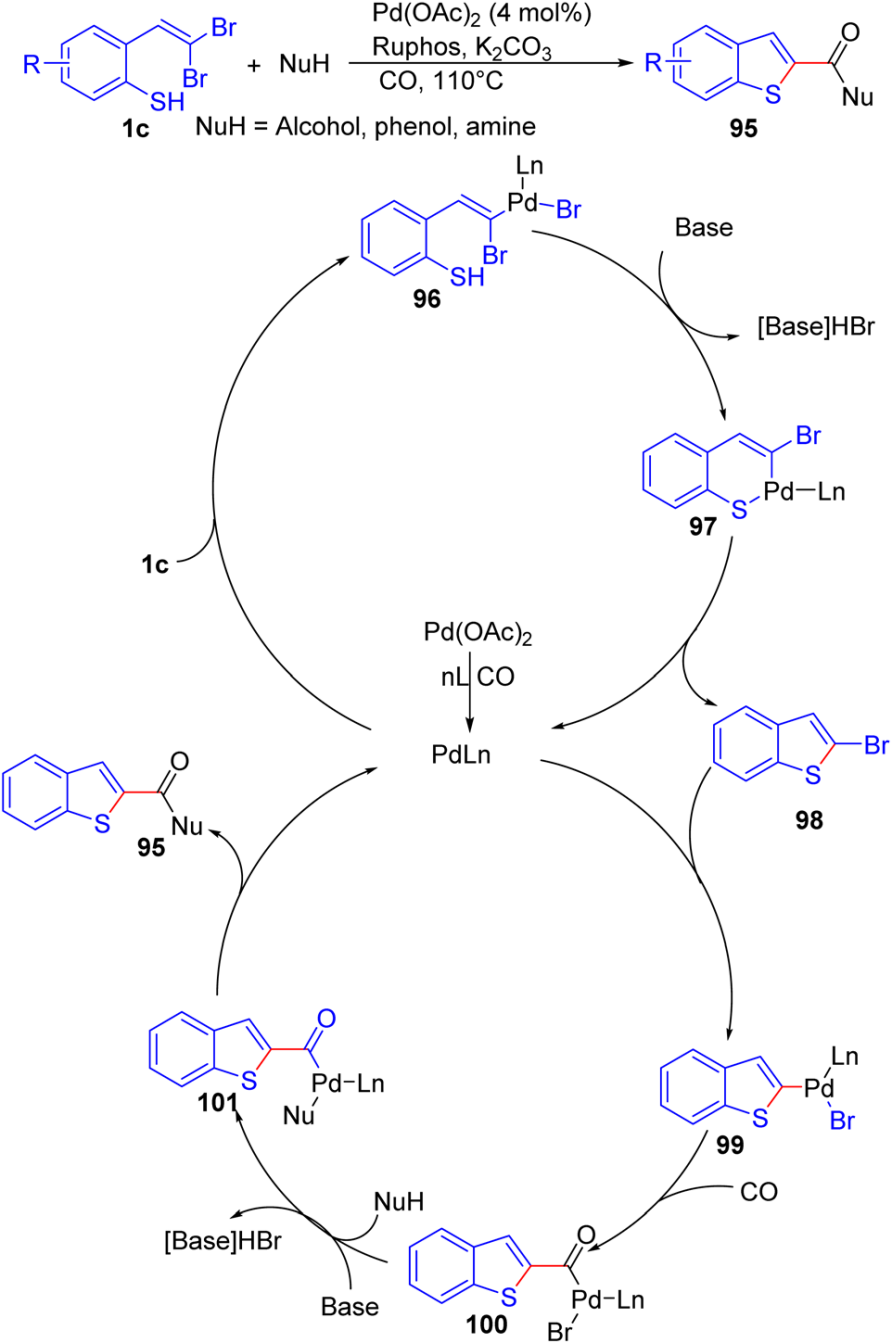
Synthesis of 2-carbonylbenzo[*b*]thiophene.

Zeng and Alper documented a highly selective synthetic route to 2-carbonylbenzo[*b*]thiophene scaffolds 95 starting from *o*-substituted *gem*-dibromoolefins 1c with alcohol, phenol and amine as nucleophiles and carbon monoxide in 2011 (Scheme 22).^40^ Their methodology involves palladium-catalyzed intramolecular C–S coupling/intermolecular carbonylation cascade sequences utilizing a Pd(OAc)_2_/2-dicyclohexylphosphino-2′,6′-diisopropoxybiphenyl (RuPhos) catalytic system with K_2_CO_3_ acting as the base. A proposed mechanism for the formation of 2-carbonylbenzo[*b*]thiophenes 95 is illustrated in Scheme 22. The process commences with the oxidative addition of 1c to the *in situ* generated pd^0^ species, resulting in palladium complex 96. Subsequently, base-catalyzed intramolecular cyclization leads to the formation of palladacycle 97. Reductive elimination of 97 delivers intermediate 2-halobenzo[*b*]thiophene 98 and regenerates the pd^0^ species. The subsequent oxidative addition of 98 to pd^0^ species forms complex 99, followed by CO insertion into the carbon–palladium bond, giving rise to intermediate 100. Aroylpalladium complex 100 is converted to intermediate 101 by base. The reductive elimination of 101 furnishes the final product 95 and regenerates the active pd^0^ species.

**Scheme 23:**
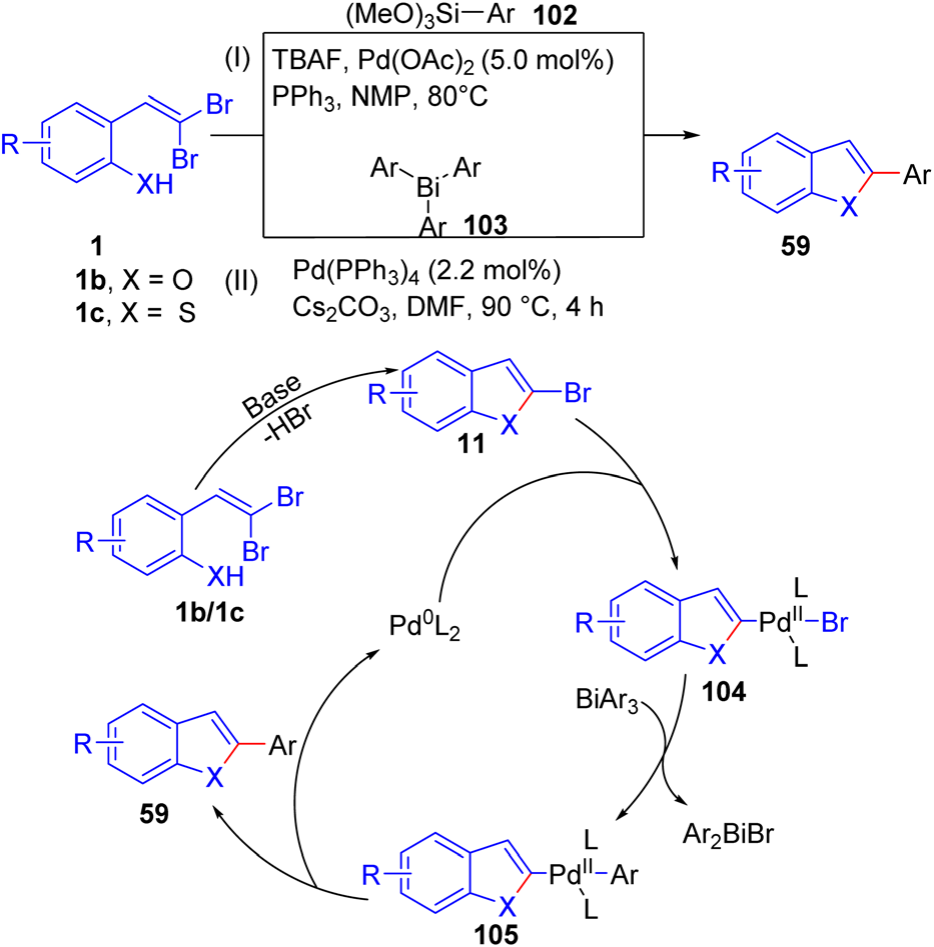
Synthesis of 2-arylbenzofurans(thiophenes).

In addition, synthesis 2-arylbenzofurans/thiophenes 59*via* tandem elimination–intramolecular addition–Hiyama reaction of *o*-substituted *gem*-dibromoolefins 1b/1c and organosilanes 102 in the presence of Pd(OAc)_2_/PPh_3_ as metal catalyst system with TBAF serving as an oxidant was developed by Wang *et al.* (Scheme 23(I)).^41^ In this transformation, the presence of electron-donating groups at the *para*-position generally led to higher yields. The reaction conditions proved effective for a range of functional groups on the benzene ring, demonstrating the versatility of this tandem reaction. In addition, the Rao group reported a tandem chemoselective synthesis of 2-arylbenzofurans 59 from *o*-substituted *gem*-dibromoolefins 1b/1c under palladium-catalyzed conditions, involving three consecutive coupling reactions with triarylbismuth reagents 103, resulting in high yields (Scheme 23(II)).^42^ The optimal conditions involved using *o*-substituted *gem*-dibromoolefins 1b/1c with BiAr_3_103 in the presence of Pd(PPh_3_)_4_ and Cs_2_CO_3_ as the base in DMF under heating conditions, this setup provided the best yield of 75%. Other bases and solvents were less effective. This method demonstrates site-selective and chemoselective tandem couplings, providing diverse functionalized 2-arylbenzofurans 59 in a one-pot operation. The authors proposed this mechanism based on several screening and control studies, starting with the base-mediated cyclization of *o*-substituted *gem*-dibromoolefins 1f to form 11. This intermediate 11 then undergoes cross-coupling with the bismuth reagent 103 under palladium-catalyzed conditions to produce 2-arylbenzofuran 59. Notably, 2-bromobenzoheterocycle 11 can form without a metal catalyst.

**Scheme 24:**
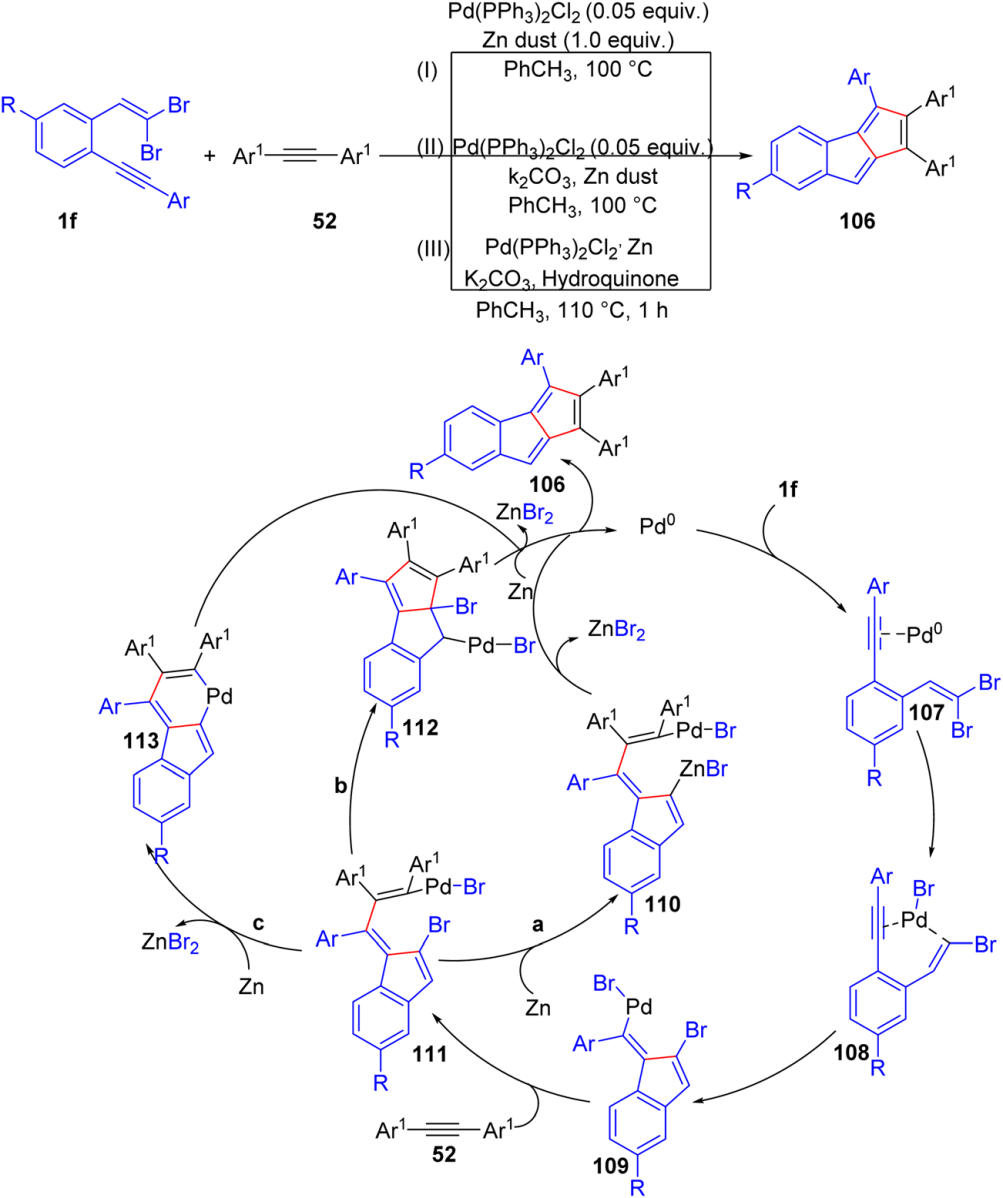
Synthesis of monoannelatedpentalenes.

Meanwhile, Rivera-Fuentes and their group developed a facile method to access monoannelated pentalenes 106*via* cascade carbopalladation reaction between alkynes 52 and *o*-substituted *gem*-dibromoolefins 1f (Scheme 24(I)).^43^ This protocol effectively provides access to a range of pentalene derivatives 106 that were previously challenging to prepare. A key advantage of this methodology lies in the accessibility of the starting materials, which are either commercially available or can be synthesized in a few steps following well-established protocols. They proposed a catalytic cycle (Scheme 24) initiated by coordination of palladium to the alkyne moiety of *o*-substituted *gem*-dibromoolefins 1f. Following, the intermediate 108 is formed from the oxidative addition of Pd^0^ to the C–Br bond. The subsequent intramolecular carbopalladation at the alkyne leads to formation a transient fulvene 109, which undergoes reaction with alkyne 52, affording intermediate 110. Three plausible termination routes were hypothesized to deliver desired product 106 by authors: (a) oxidative addition of zinc to the C–Br bond 111, followed by an intramolecular Negishi-type sequence. (b) Intramolecular carbopalladation at the fulvene 110, followed by zinc-assisted reductive elimination. (c) Two-electron reduction of the Pd(ii) intermediate 110 by zinc, a second oxidative addition, formation of a palladacycle 113, and reductive elimination. A short time later, Diederich and co-workers also extended this methodology to improve its yield (Scheme 24(II)).^44^ Better reaction efficiency was achieved by incorporating two equivalents of K_2_CO_3_, which significantly increased the yield of benzopentalene 106 from 42% to 68%. Maintaining a high excess of diarylacetylene 52 (20 equivalents) was found to be crucial as well, as decreasing it to 10 equivalents reduced the yield to 48%. It should be noted, this methodology was further extended to synthesize pentalenes 106 with novel fusion patterns *via* intramolecular carbopalladation, achieved by attaching an additional alkyne 52 to the *o*-substituted *gem*-dibromoolefins 1f. Meanwhile, very recently, Gazdag *et al.* also utilized Rivera-Fuentes and their group's method^45^ to generate a wide variety of monobenzopentalenes derivatives 106 and reported how different substituents can affect their photophysical properties (Scheme 24(III)). A series of substituted monobenzopentalenes 106 were synthesized in 18–85% yields, showcasing the potential of the cascade reaction for forming three new C–C bonds. The methodology proved versatile, enabling the synthesis of molecules with electron-donating and electron-withdrawing functional groups directly attached to the benzopentalene cores 106 and substituents on the aryl groups.

**Scheme 25:**
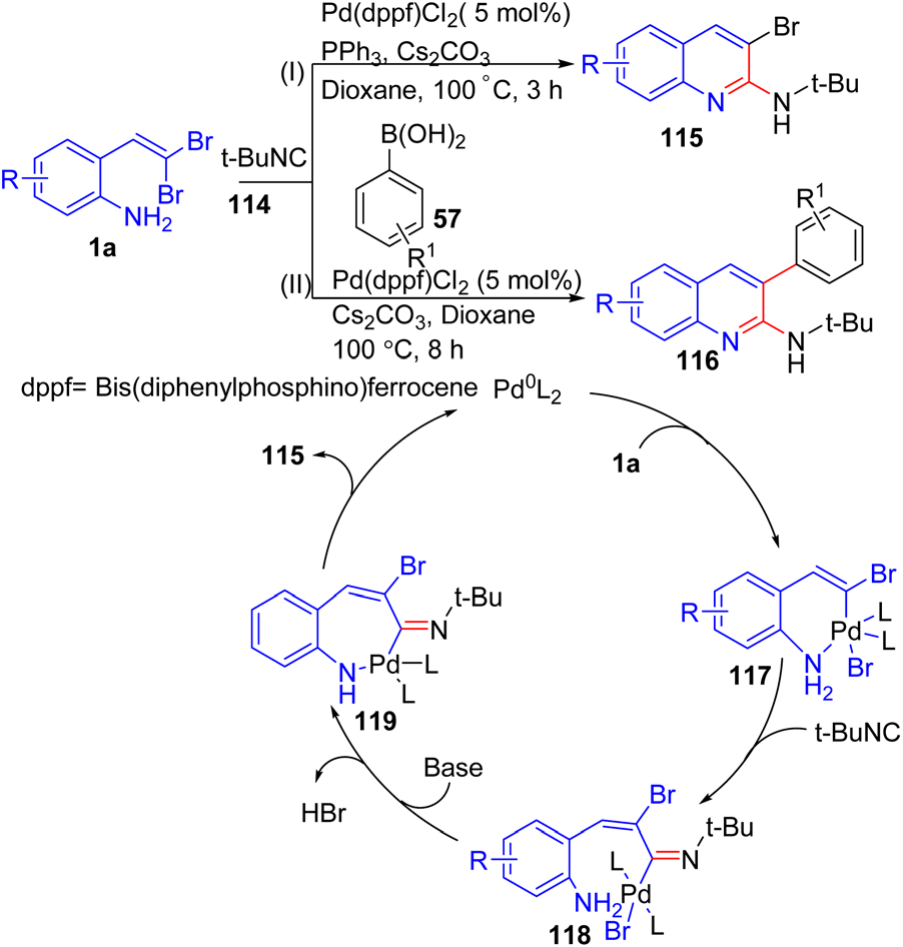
Synthesis of 2-amino-3-bromoquinolines and 3-aryl-2-aminoquinolines.

Jiang and co-workers introduced a palladium-catalyzed method for synthesizing 2-amino-3-bromoquinolines 115*via* isocyanide 114 insertion/intramolecular cyclization of *ortho*-substituted *gem*-dibromoolefins 1a (Scheme 25(I)).^46^ Conducted in 1,4-dioxane at 100 °C, the reaction delivers the desired quinoline cyclization and as well as reductive elimination, yielding the 2-amino-3-bromoquinoline products 115 and the regenerates the active Pd^0^ species. The reaction scope covers various *o*-substituted *gem*-dibromoolefins 1a with both electron-donating and electron-withdrawing substituents on the aromatic ring, producing polyhalogenated quinolines 115. This method was further extended to a one-pot process for synthesizing 3-substituted-2-aminoquinolines 115 through Suzuki, Sonogashira, or Heck coupling reactions, providing a scalable approach to a wide range of quinoline derivatives. When, their group performed this reaction with arylboronic acids 57 led to formation of 3-aryl-2-aminoquinolines 116 (Scheme 25(II)).^47^ This reaction follows a mechanism similar to their previous work, involving palladium-catalyzed isocyanide insertion and intramolecular cyclization of *o*-substituted *gem*-dibromoolefins 1a and finally, Suzuki coupling with arylboronic acids 57. The reaction scope of this method was notably broad, with various *o*-substituted *gem*-dibromoolefins 1a and arylboronic acids 57 successfully participating in the reaction. Substituents on the *o*-substituted *gem*-dibromoolefins 1a, whether electron-donating or electron-withdrawing, were well tolerated, offering flexibility in designing functionalized quinolines. A key observation was that the nature of the arylboronic acids 57 significantly impacted yields. Electron-donating groups, such as methoxy or methyl, consistently provided higher yields, while halogen groups, like fluoro or chloro, led to slightly reduced efficiencies. An intriguing aspect of the scope was the method's ability to accommodate heteroaryl boronic acids 57. For example, 3-thienylboronic acid participated smoothly in the reaction, delivering the corresponding 3-thienyl-2-aminoquinoline 116 in high yield, showcasing the adaptability of the method for synthesizing heterocyclic quinolines.

**Scheme 26:**
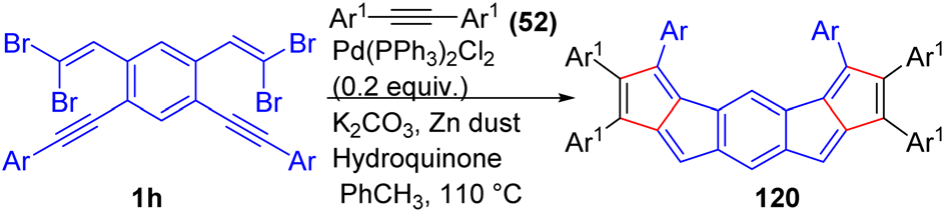
Synthesis of π-extended bispentalene derivatives.

The synthesis of π-extended bispentalene derivatives 120 with different fusion patterns and functional groups on the peripheral phenyl groups through a modified double carbopalladation cascade reaction between acetylenes 52 and bis(*gem*-dibromoolefins) 1h with the formation of six C–C bonds during the one-pot reaction was reported by Diederich and co-workers (Scheme 26).^48^ This research team also investigated the effects of antiaromatic subunits within [4*n* + 2] π-systems by synthesizing bispentalenes possessing [4*n* + 2] π-electron perimeters and antiaromatic characteristics. It should be noted, when they investigated reaction in the presence of hydroquinone, significantly improved the reaction outcome, forming six new C–C bonds in a one-pot cascade reaction. Notably, this approach also allowed for a substantial decrease in the required acetylene reagent, from 40 equivalents to just 6 equivalents.

**Scheme 27:**
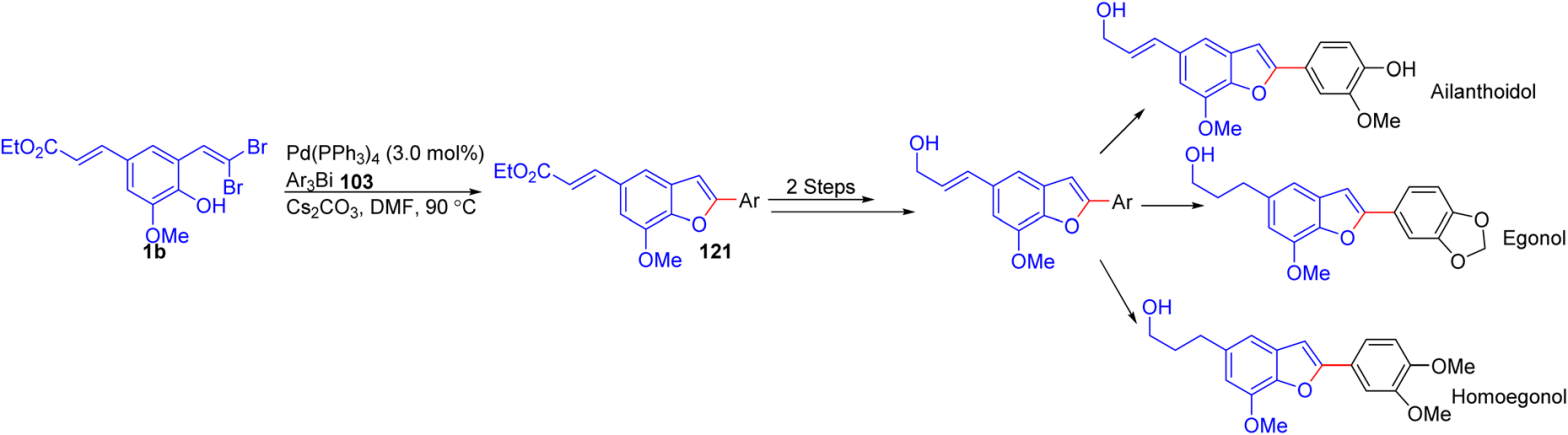
Preparation of several benzofuran-based natural products.

In a 2016 study, Rao and Murty developed a concise synthetic strategy for the preparation of several benzofuran-based natural products, including ailanthoidol, egonol, and homoegonol, among others (Scheme 27).^49^ The key methodology employed was a Pd-catalyzed domino cyclization/coupling reaction utilizing *o*-substituted *gem*-dibromoolefins 1b and triarylbismuth reagents 103. This one-pot process efficiently generated the core benzofuran skeleton, which was subsequently functionalized to yield the target natural products. The reported methodology proved tolerant, accommodating a range of *o*-substituted *gem*-dibromoolefins 1b and various triarylbismuth reagents 103 to synthesize 2-arylbenzofuran derivatives 121 in high yields.

**Scheme 28:**
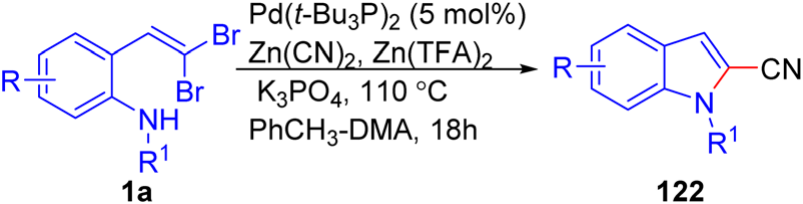
Synthesis of 2-cyanoindoles.

Lautens *et al.* have introduced a highly effective Pd-catalyzed method for synthesizing 2-cyanoindoles 122 from *o*-substituted *gem*-dibromoolefins 1a and Zn(CN)_2_ as the cyanide source in the presence of Zn(TFA)_2_ to enhance catalytic performance in toluene (Scheme 28).^50^ Notably, in the absence of DMA as a cosolvent, the starting material was fully consumed, resulting primarily in cyclization with 2-bromoindole 29 as the major product and no cyanation occurring. The optimized methodology showed to be compatible with a range *o*-substituted *gem*-dibromoolefins 1a bearing electron-donor and electron-withdrawing groups on the aryl rings, yielding the respective products 122 in moderate to good yields.

**Scheme 29:**
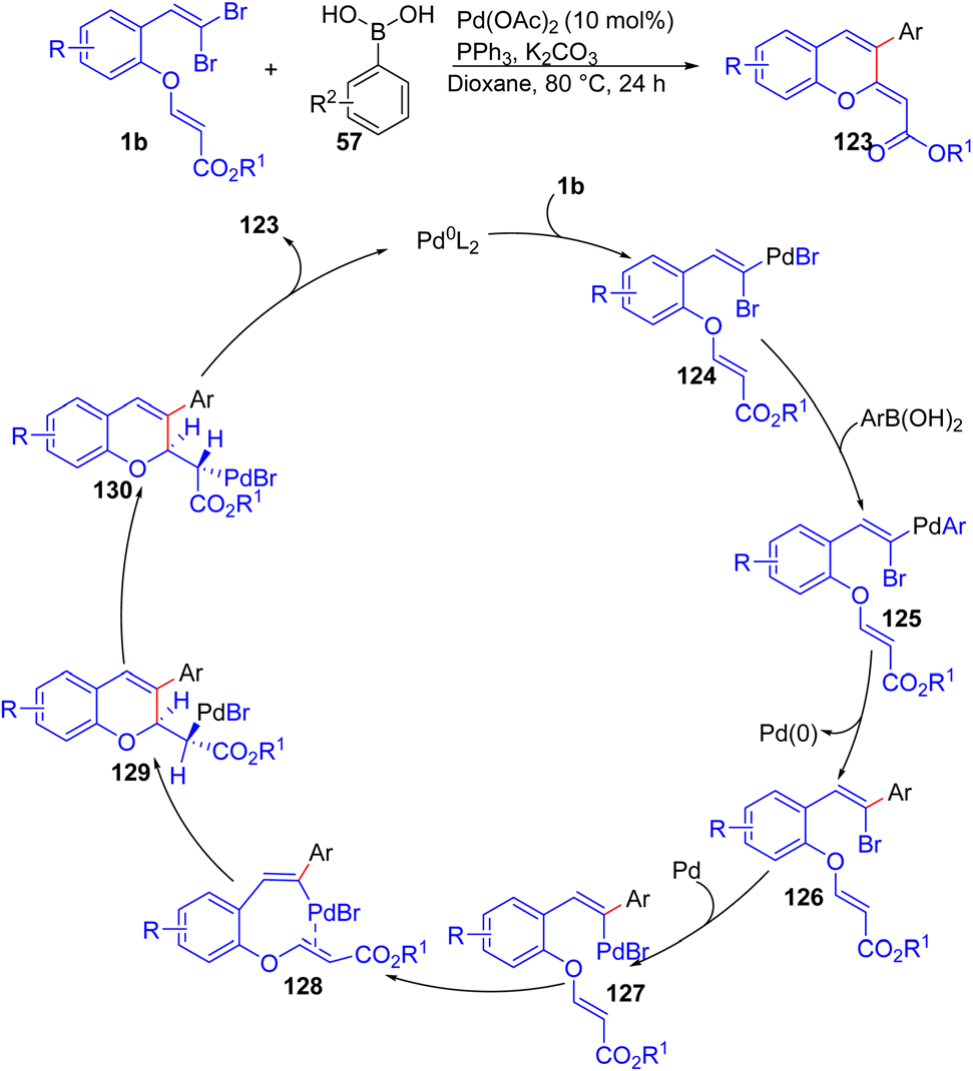
Synthesis of functionalized 2*H*-chromenes.

A 2018 study by Song *et al.* presents a novel synthesis of functionalized 2*H*-chromenes 123*via* Pd-catalyzed cascade reactions of *o*-substituted *gem*-dibromoolefins 1b with aryl boronic acids 57, combining two C–C bond-forming reactions *via* intermolecular Suzuki coupling and intramolecular Heck coupling in a one-pot reaction (Scheme 29).^51^ By this novel and simple approach, the authors prepared variety of desired products 123 in moderate to good yields. The formation of 123 from 1b and 57 was proposed to involve the oxidative addition of Pd^0^ into the C–Br bond of 1b, forming intermediate 124 which is converted to intermediate 125 by transmetalation with 57. Then, intermediate 125 undergoes reductive elimination to deliver 126 and regenerate Pd^0^. Another oxidative addition into the vinyl C–Br bond of 126 forms 127, leading to intermediates 128 and then 129 through palladium coordination and insertion reaction. Rotation around the Cα–Cβ bond in intermediate 129 produces 130, which, after HPdBr elimination, affords 123 and regenerates Pd^0^.

**Scheme 30:**
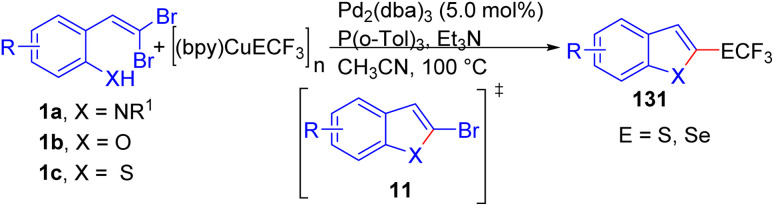
Synthesis of 2-trifluoromethylthio(seleno)-substituted benzofused heterocycles.

In 2019, Zhang and Weng pioneered a general strategy for synthesizing 2-trifluoromethylthio(seleno)-substituted benzofused heterocycles 131 through reaction of *o*-substituted *gem*-dibromoolefins 1a/1b/1c with (bpy)CuSCF_3_ or with [(bpy)CuSeCF_3_]_2_ in the presence of Pd_2_(dba)_3_ and P(*o*-Tol)_3_ as the catalyst and ligand, respectively in acetonitrile solvent at 100 °C for 24 hours (Scheme 30).^52^ They suggested a mechanism involving palladium-catalyzed intramolecular cross-coupling of 1a/1b/1c to form 2-bromobenzoheterocycle 11 as key intermediate, which then undergoes trifluoromethylthiolation with (bpy)CuSCF_3_ or with [(bpy)CuSeCF_3_]_2_ to yield the desired product 131. In addition, this strategy was applied to prepare 2-trifluoromethylthiolated benzothiophenes and indoles.

**Scheme 31:**
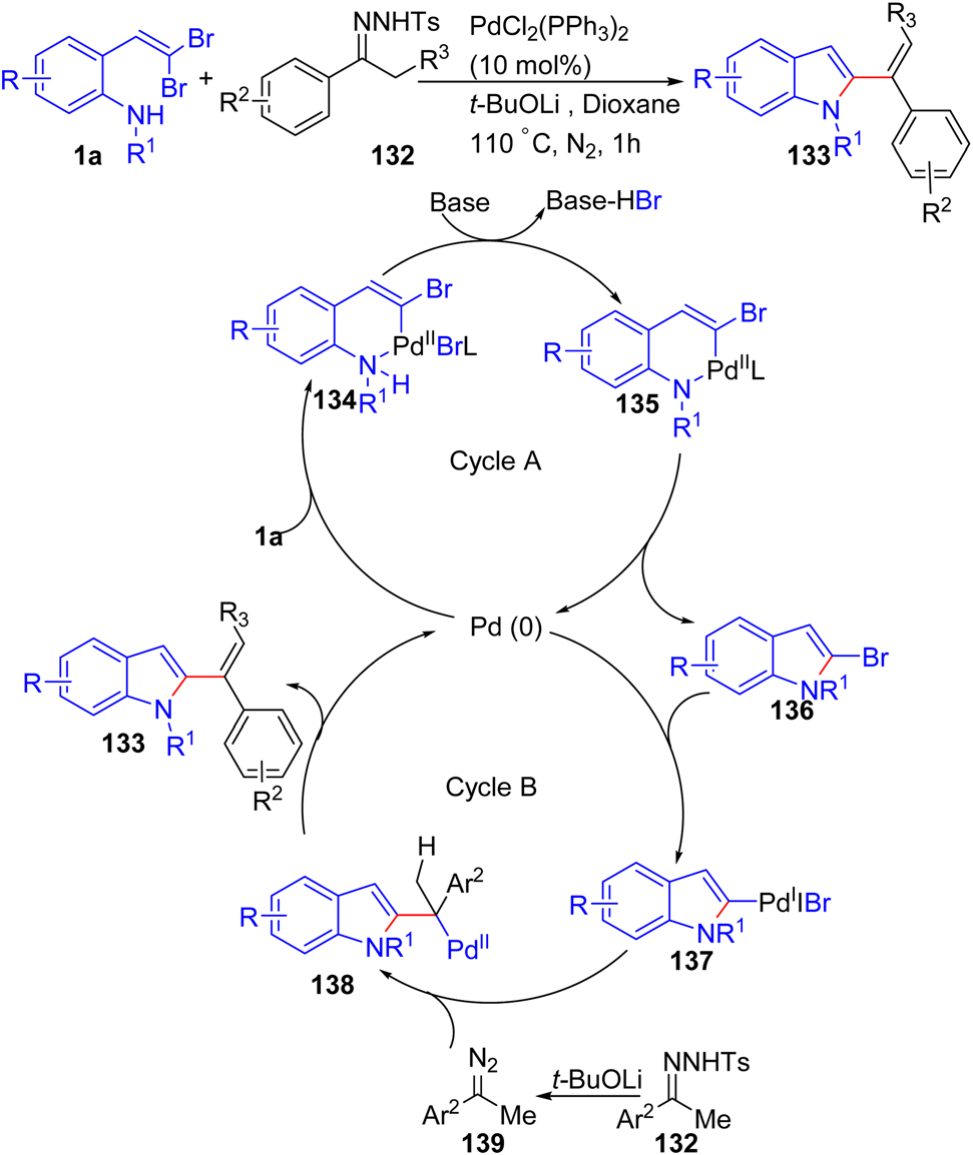
Synthesis of 2-(1-phenylvinyl)-indoles.

Liu *et al.* described a novel palladium-catalyzed intermolecular coupling reaction of *o*-substituted *gem*-dibromoolefins 1a and *N*-tosylhydrazones 132 to efficiently construct 2-(1-phenylvinyl)-indoles 133, obtaining indoles bearing 1,1-disubstituted alkenes in one step with a short reaction time, a broad substrate scope and high yields (Scheme 31).^53^ The reaction begins with cycle A, where the oxidative addition of palladium to *o*-substituted *gem*-dibromoolefins 1a forms the palladacycle 134. Deprotonation of aniline then produces palladium complex 135, which undergoes reductive elimination to give compound 136. The authors also reported that this compound 136 can be observed by TLC and GC-MS but disappears by the end of the reaction. In cycle B, the oxidative addition of compound 136 to palladium affords palladium(ii) complex 137, which is converted to alkylpalladium species 138 by a carbene intermediate from diazo compound 139. Finally, β-hydrogen elimination of complex 138 delivers the desired product, 2-(1-phenylvinyl)-indole 133.

**Scheme 32:**
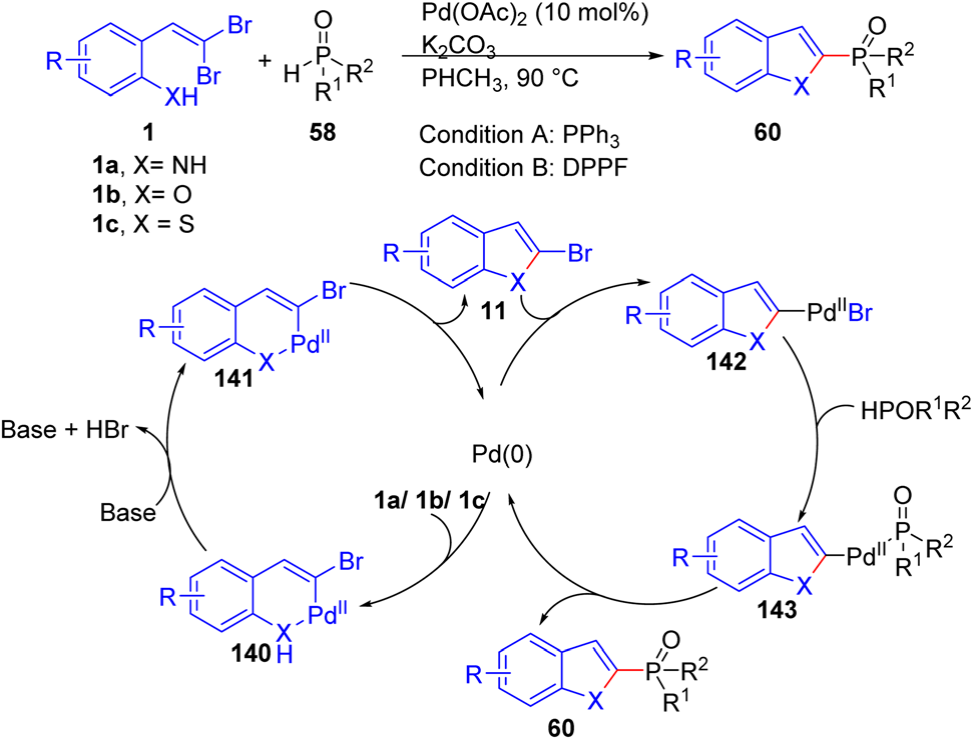
Synthesis of phosphorylated heteroaromatics.

Chen and colleagues recently documented the synthesis of phosphorylated heteroaromatics 60*via* palladium-catalyzed domino cyclization/phosphorylation of *o*-substituted *gem*-dibromoolefins 1a/1b/1c, when the X = O, S is in the starting material, suitable ligand is PPh_3_ under optimized reaction conditions, while DPPF was selected as appropriate ligand for X = NH in this transformation (Scheme 32).^54^ A series of control experiments were conducted to propose a plausible mechanism. The process begins with the oxidative addition of Pd^0^ to the C–Br bond of *o*-substituted *gem*-dibromoolefins 1a/1b/1c, followed by coordination with the XH group, forming the intermediate 140. Subsequent deprotonation of XH leads to the formation of the six-membered palladacycle complex 141. Then palladacycle complex undergoes reductive elimination, producing intermediate 11 and releasing the Pd^0^ species.

Oxidative addition of Pd^0^ to the C–Br bond of 11 delivers the intermediate 142. Intermediate 143 is generated by ligand exchange with HP(O)R^1^R^2^. Finally, intermediate 143 undergoes reductive elimination, affording the phosphorylated heteroaromatics 60.

**Scheme 33:**
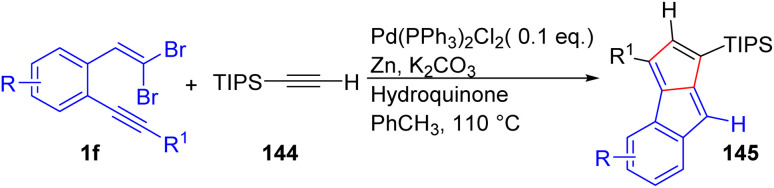
Monoareno-pentalenes with two olefinic protons.

Recently, based on the same methodology used by Rivera-Fuentes^43^ and their group, Mayer and London attempted to access monoareno-pentalenes 145 that have an olefinic H on each 5-membered ring through regioselective carbopalladation cascade reaction between *o*-substituted *gem*-dibromoolefins 1f and TIPS-acetylene 144 (Scheme 33).^55^ Overall, the authors proposed these novel pentalenes, possessing two olefinic protons, could serve as valuable experimental tools for further exploring magnetic (anti)aromaticity effects.

**Scheme 34:**
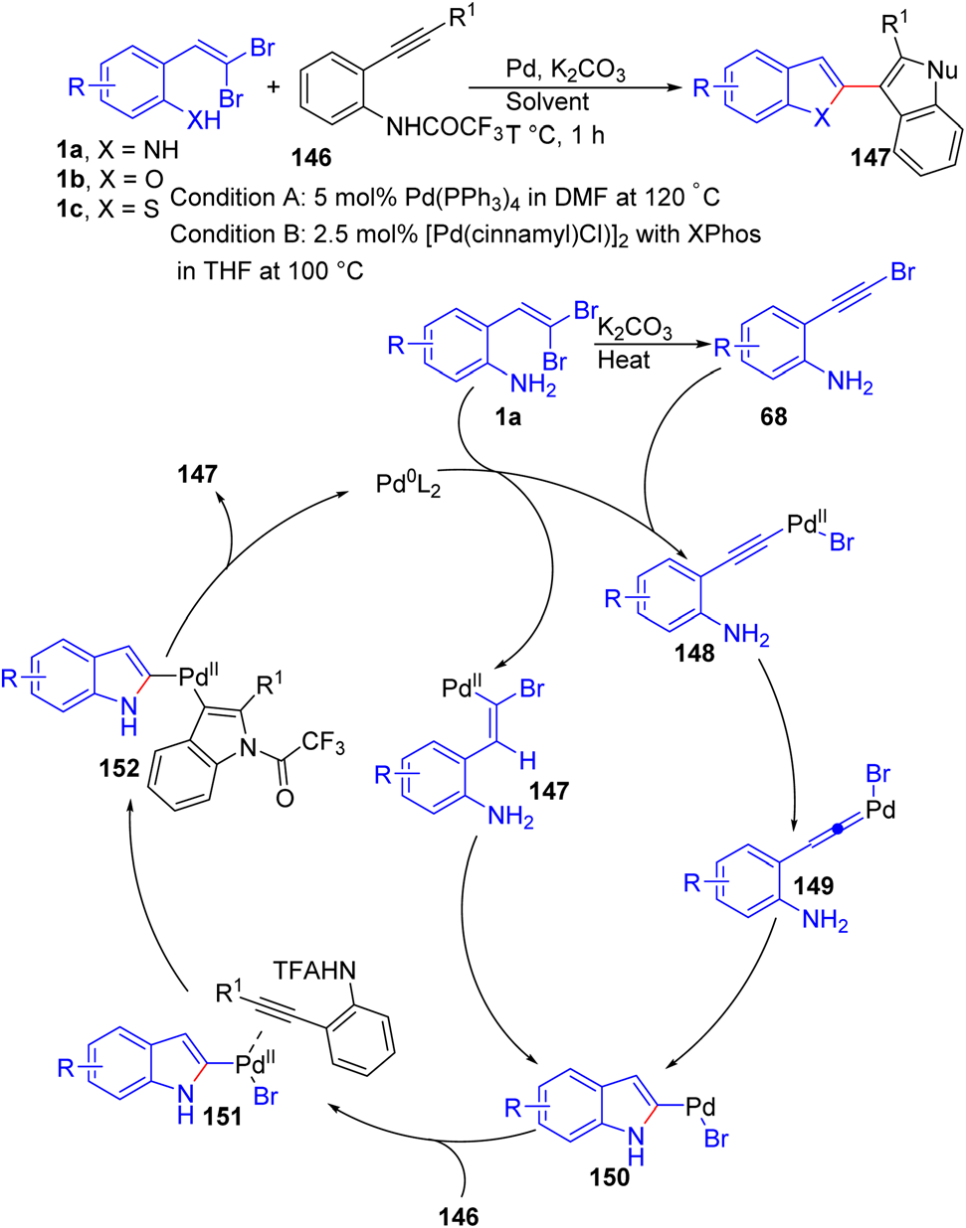
Formation of bis-heterocycles.

Lautens and his team recently reported a broad-scope method for synthesis bis-heterocycles 147. This new method involves a palladium-catalyzed tandem C–N coupling/Cacchi reaction starting from *o*-substituted *gem*-dibromoolefins 1a/1b/1c, generating two heterocycle rings 147 (Scheme 34).^56^ The reaction between dibromoolefines 1a/1b/1c and alkyne-tethered anilines 146, employing 5 mol% Pd(PPh_3_)_4_, K_2_CO_3_ in DMF (0.1 M) at 120 °C, gave desired products 147 with good to excellent yields. It should be noted that, having the alkyne-tethered aniline 146 as a trifluoroacetamide was necessary for high yields. In addition, use of a dibromoolefinic phenol/thiophenol instead of aniline provided the corresponding benzofuran/benzothiophene bis-heterocycles. Notably, when, dibromoolefinic phenols as starting material were used, they employed new condition including [Pd(cinnamyl)Cl]_2_ as catalyst and 2-dicyclohexylphosphino-2′,4′,6′-triisopropylbiphenyl (XPhos) as ligand in THF at 100 °C. The mechanism depicted in Scheme 34 was suggested for this transformation, that involves formation of a bromoalkyne 68 as a competent intermediate in the reaction.

**Scheme 35:**
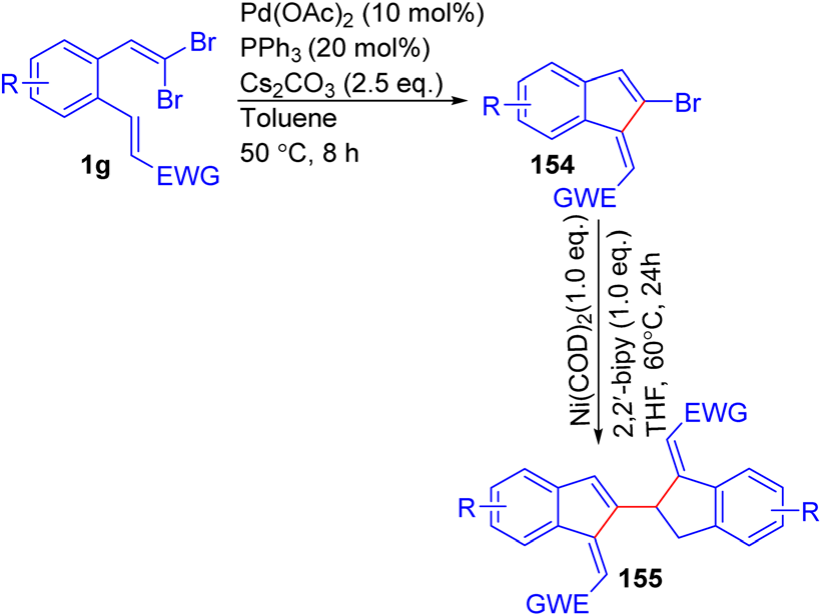
Synthesis of bis-benzofulvenes.

In 2023, Ganesh *et al.* described the synthesis of bis-benzofulvenes 155 through a palladium-catalyzed intramolecular Heck coupling followed by Ni-mediated C(sp^2^)–Br dimerization (Scheme 35).^57^ The reaction was designed to explore the optoelectronic properties of the resulting bis-benzofulvenes, which are of interest due to their extended conjugation and potential applications in functional materials. During their exploration of reaction conditions, Ganesh's group found that the yield was significantly affected by the choice of phosphine ligand and the conditions for the Ni-mediated dimerization. Bulky phosphines, like PtBu_3_, generally led to poor yields, while smaller cone angle ligands such as triphenylphosphine (PPh_3_) improved yields, reaching up to 89% for bromobenzofulvene 154. This adaptability highlights the method's potential for creating structurally varied bis-benzofulvenes 155 with wide applications, particularly in the development of functional materials with tailored electronic properties. The reaction maintained high efficiency under mild conditions, making it applicable for a broad range of synthetic applications, particularly in the field of optoelectronic material.

**Scheme 36:**
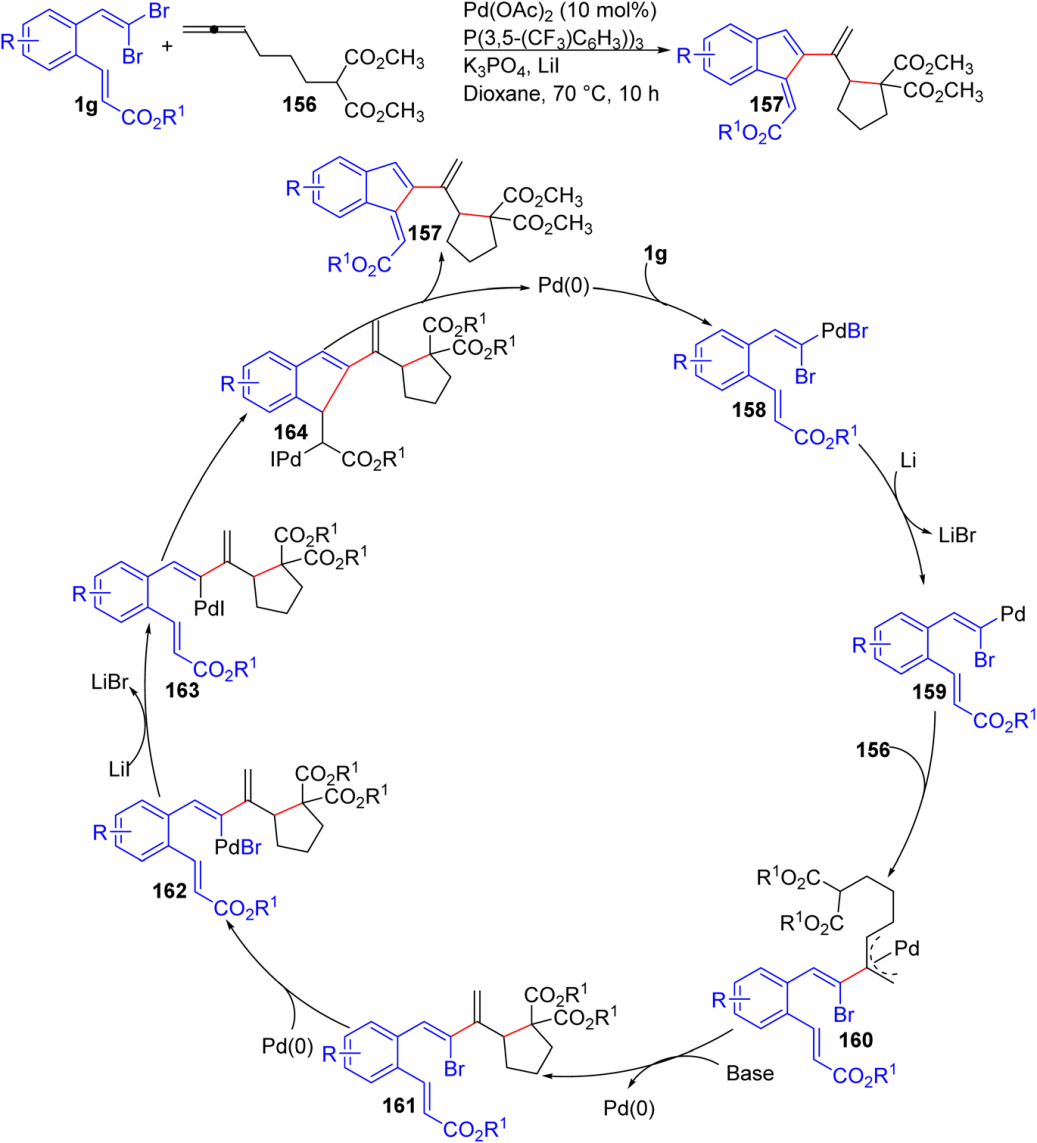
Pd-catalyzed bicyclization of *ortho*-substituted *gem*-dibromoolefins.

Zhou *et al.* reported a Pd-catalyzed bicyclization of o-substituted *gem*-dibromoolefins 1g with allenyl malonates 156, synthesizing bicyclic compounds 157 *via* a one-pot cascade reaction (Scheme 36).^58^ The reaction proceeds using Pd(OAc)_2_ as the catalyst, a phosphine ligand (P[3,5-(CF_3_)_2_C_6_H_3_]_3_), and K_3_PO_4_ in 1,4-dioxane, with LiI as an additive, constructing three C–C bonds and two new rings in one single step. The process begins with oxidative addition of Pd^0^ into the C–Br bond of the o-substituted *gem*-dibromoolefins 1g, forming a vinylpalladium intermediate 158. Halide exchange generates a reactive intermediate 159, which undergoes intermolecular carbopalladation with the allenyl malonate 156 to yield a π-allylpalladium species 160. Next, internal cyclization of 160 affords intermediate 161 which is converted to intermediate 162 by oxidative addition. Subsequent halide exchange and intramolecular Heck reaction lead to ring closure, and β-hydride elimination regenerates the Pd^0^ catalyst while forming the final bicyclic product 157. Control experiments also confirmed the sequential nature of the reported reaction. Furthermore, the reported methodology proved to be highly tolerant. Various *o*-substituted *gem*-dibromoolefins 1g bearing electron-donating groups, such as methyl and methoxy substituents, were effectively transformed into the corresponding bicyclic products 157 with high efficiency. Substrates with electron-withdrawing groups, including nitro, ester, and trifluoromethyl functionalities, also reacted smoothly under the optimized conditions, yielding the desired compounds 157 in excellent yields. Moreover, the reaction conditions accommodated not only symmetrical but also unsymmetrical allenyl malonate derivatives 156, allowing for the synthesis of structurally diverse bicyclic products 157. Heteroatom-containing allenes, such as those derived from allenic alcohols or amines, also participated successfully in the reaction, producing five-membered heterocyclic products in moderate to good yields.

**Scheme 37:**
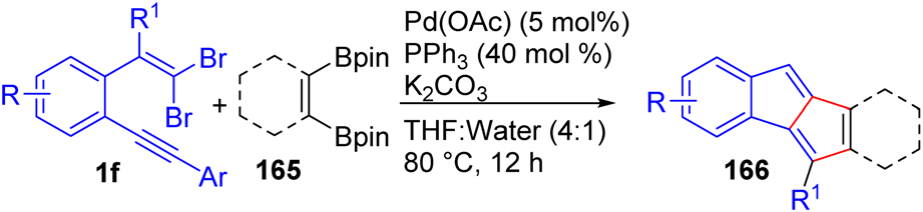
Synthesis of unsymmetrical dibenzopentalenes.

Very recently, Ganesh and co-workers have developed Pd(0)-catalyzed cascade Suzuki/carbopalladation strategy for the efficient synthesis of diverse unsymmetrical dibenzopentalenes 166 from *gem*-dibromo olefins 1f and benzene-1,2-diboronic esters 165 (Scheme 37).^59^ Optimization studies revealed that Pd(OAc)_2_ (10 mol%) in combination with PPh_3_ (40 mol%) and K_2_CO_3_ in THF/H_2_O (4 : 1) was crucial for achieving efficient cascade coupling, with water serving as an essential cosolvent to improve K_2_CO_3_ solubility. Under these conditions, a wide variety of *gem*-dibromo olefins bearing electron-donating, electron-withdrawing, and heteroaryl substituents were smoothly transformed into unsymmetrical dibenzopentalenes 166 in 39–84% yields. In addition, the authors further examined photophysical and electrochemical studies highlighting the unique optoelectronic properties of these antiaromatic scaffolds. UV-vis spectra revealed weak, broad absorptions across 400–700 nm, consistent with symmetry-forbidden HOMO → LUMO transitions, whereas cyclic voltammetry of representative dibenzopentalenes, supported by DFT and TD-DFT calculations, confirmed characteristic redox behavior.

### 2.3. Rhodium-catalyzed cyclization

Rhodium is recognized as modern catalyst in chemistry due to high efficiency and selectivity in various chemical reactions and serves as a suitable catalyst in diverse fields, such as synthesis of pharmaceuticals and fine chemicals.^60^

**Scheme 38:**
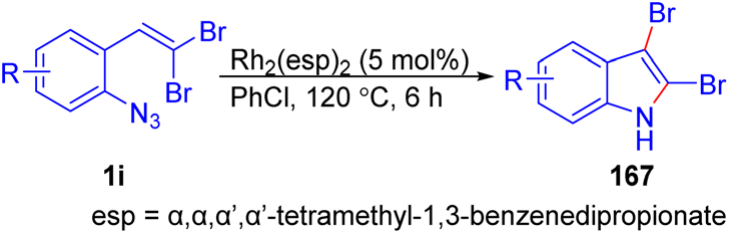
Synthesize of 2,3-dibromoindoles.

Recently, Hata *et al.* reported a Rh-catalyzed intramolecular cyclization and rearrangement of *o*-substituted *gem*-dibromoolefins 1i to synthesize 2,3-dibromoindoles 167 (Scheme 38).^61^ When, they carried out reaction of *o*-substituted *gem*-dibromoolefins 1i using Rh_2_(esp)_2_ as catalyst, giving 2,3-dibromoindoles 167 in 51–74% yields. This reaction is performed *via* the intramolecular cyclization of rhodium nitrene generated *in situ* and the rearrangement of one halogen group.

### 2.4. Dual-metal-catalyzed cyclization

Dual-metal-catalyzed reactions involving *o*-substituted *gem*-dibromoolefins have emerged as powerful synthetic strategies, combining the complementary properties of two distinct metal catalysts or two subsequent different metal-catalyzed reactions to achieve transformations unattainable or inefficient with a single metal system. These methodologies leverage synergistic effects between metals to facilitate sequential catalytic cycles or tandem processes. By capitalizing on distinct reactivities, dual-metal catalysis enables intricate cyclizations and functionalizations under relatively mild conditions. This section discusses recent progress in employing dual-metal catalysis for transformations of *o*-substituted *gem*-dibromoolefins.^62^

**Scheme 39:**
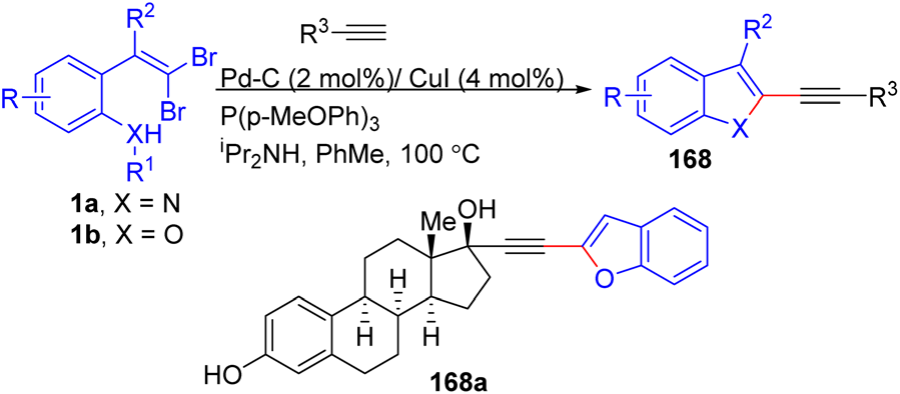
Synthesis of 2-alkynyl indoles and benzofurans.

Another perfect example of utilizing *o*-substituted *gem*-dibromoolefins 1a/1b in tandem Ullman/Sonogashira coupling was reported by Nagamochi *et al.* (Scheme 39).^63^ Using readily available *o*-substituted *gem*-dibromoolefins 1a/1b and terminal alkynes, they synthesized a series of 2-alkynyl indoles and benzofurans 168*via* CuI-catalyzed intramolecular Ullman and Pd/C-catalyzed Sonogashira coupling cascades. Various aromatic and aliphatic terminal alkynes with different electronic properties using *o*-substituted *gem*-dibromoolefins 1a/1b yielded 2-alkynyl indoles 168 in moderate to good yields. Altering electron-donating and electron-withdrawing groups on the dibromoolefin substrates 1a/1b did not affect the tandem.reaction's efficacy, delivering desired products 168 in 55–84% yields. Notably, the authors also successfully synthesized a complex steroid derivative with two acidic hydroxyl groups, achieving a good yield.

**Scheme 40:**
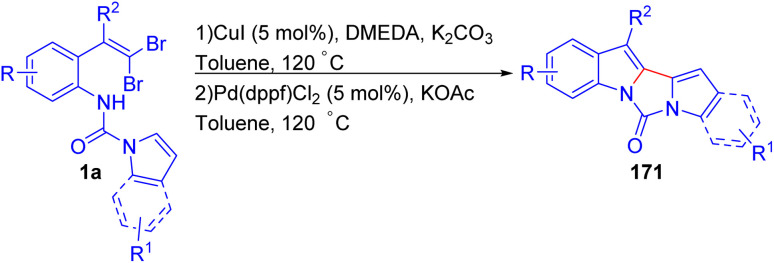
Synthesis of pyrimido[1,6-*a*]indol-1(2*H*)-one derivatives.

Bao's group reported a convenient one-pot protocol for synthesizing pyrimido[1,6-*a*]indol-1(2*H*)-one derivatives 170 through a nucleophilic addition/Cu-catalyzed *N*-arylation/Pd-catalyzed C–H activation sequence (Scheme 40).^64^ The methodology employes *o*-substituted *gem*-dibromoolefins 1j and *N*-alkyl-anilines 169 as starting materials, which underwent a three-step process to afford the desired indole-fused frameworks 170 in moderate to good yields. Initial studies identified CuI with *N*,*N*′-dimethylethylenediamine (DMEDA) and K_2_CO_3_ as optimal for the *N*-arylation step, and Pd(dppf)Cl_2_ with KOAc for the direct arylation conditions. The authors found that the reaction scope was broad, tolerating both electron-rich and electron-deficient substituents on the *o*-substituted *gem*-dibromoolefins 1j, and producing functionalized pyrimido[1,6-*a*]indol-1(2*H*)-ones 170 with minimal steric hindrance effects. When exploring *N*-alkyl-anilines 169, electron-donating groups such as 4-Me, 4-MeO, and 3-Me on the aniline ring yielded products 170 efficiently, while electron-withdrawing groups like 4-NO_2_ and 4-Ac were detrimental, likely due to reduced nucleophilicity. The reaction's versatility extended to *N*-benzyl and *N*-naphthyl derivatives, demonstrating applicability to more sterically demanding substrates.

**Scheme 41:**
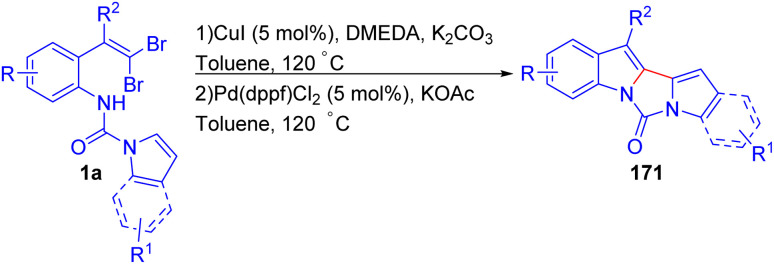
Synthesis of unsymmetrical 2,2′-biindolyl derivatives.

Bao *et al.* developed a one-pot two-step strategy for the synthesis of unsymmetrical 2,2′-biindolyl derivatives 171*via* a sequential Cu-catalyzed *N*-arylation and Pd-catalyzed direct arylation using *o*-substituted *gem*-dibromoolefins 1a (Scheme 41).^65^ Initial optimization showed that CuI, in combination with *N*,*N′*-dimethyl ethylenediamine (DMEDA) and K_2_CO_3_, was effective for the *N*-arylation step, while Pd(dppf)Cl_2_ with KOAc promoted the subsequent direct arylation, yielding the desired products 171 in moderate to good yields. Interestingly, the reaction tolerated a range of electron-donating and electron-withdrawing substituents on the *o*-substituted *gem*-dibromoolefins 1a, and the protocol proved efficient for both sterically hindered and less hindered substrates, unlike many existing methods. This flexibility was further demonstrated by synthesizing biindolyls 171 with substituents like 4,5-diMeO and 4-Cl, 4-Br, 5-Br, as well as unsymmetrical biindolyls 171 incorporating different substituents on each indole ring. A notable finding was that when indole was replaced by pyrrole, the reaction proceeded smoothly, albeit with lower yields, extending the methodology's utility to related heterocycles. However, when both indole rings bearing electron-withdrawing substituents, the reaction was unsuccessful, possibly due to instability of the starting materials.

**Scheme 42:**
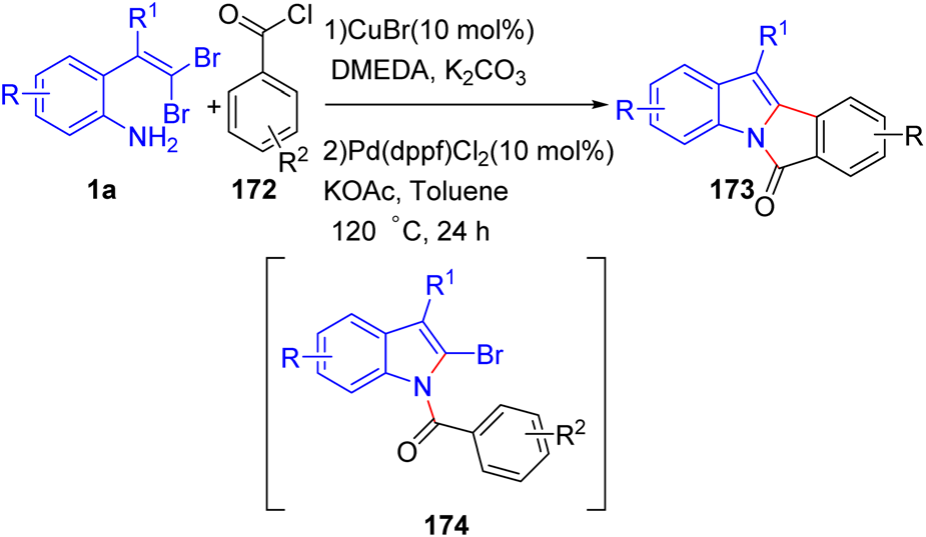
Synthesis of 6*H*-isoindolo[2,1-*a*]indol-6-ones.

In 2012, Bao and co-workers described a one-pot synthesis of 6*H*-isoindolo[2,1-*a*]indol-6-ones 173 using a similar strategy as their previous works (Scheme 42).^66^ The reaction employs *o*-substituted *gem*-dibromoolefins 1a and benzoyl chlorides 172 as starting substrates, and CuBr and Pd(dppf)Cl_2_ as a dual catalytic system. Optimization studies revealed that CuBr, in combination with DMEDA and K_2_CO_3_, is the most effective for the initial C–N coupling cyclization, leading to formation (2-bromo-1*H*-indol-1-yl)(aryl)methanones 174, while Pd(dppf)Cl_2_ and KOAc promote the subsequent C–H activation step, affording desired products 173. Electron-withdrawing groups on the aniline component were found to enhance the efficiency of the copper-catalyzed cyclization, providing high yields of the indole intermediate, while electron-donating substituents on the benzoyl chloride accelerated the palladium-catalyzed C–H activation. Notably, when *m*-methyl benzoyl chloride 172 was used, a single regioisomer was obtained demonstrating high regioselectivity in the C–H activation step. The methodology showcased broad functional group tolerance, enabling the synthesis of a diverse set of 6*H*-isoindolo[2,1-*a*] indol-6-one derivatives 173 with varied substitution patterns, suggesting its potential utility in complex molecule synthesis and pharmaceutical applications.

**Scheme 43:**
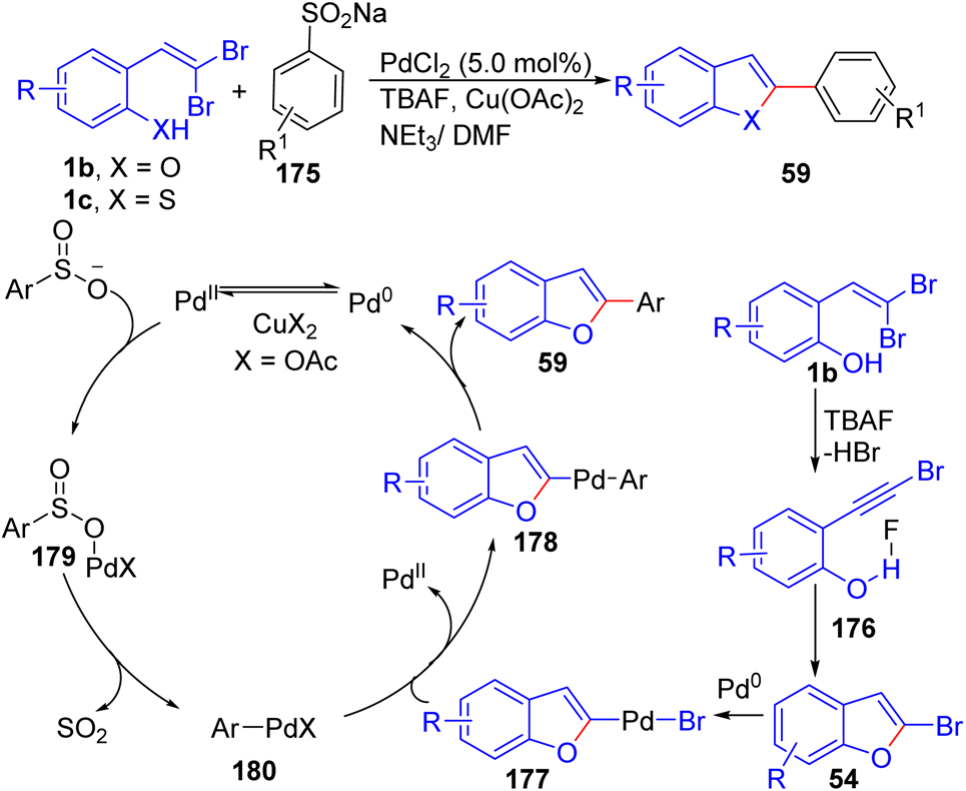
A ligand-free, one-pot synthesis of 2-arylbenzofurans(thiophenes).

A ligand-free, one-pot procedure has been developed by Wang *et al.*.for the synthesis of 2-arylbenzofurans(thiophenes) 59, through tandem elimination–cyclization–desulfitative arylation of *o*-substituted *gem*-dibromoolefins 1b/1c in the presence of sodium arylsulfinate 175, TBAF, PdCl_2_, Cu(OAc)_2_ and NEt_3_ (Scheme 43).^67^ A proposed mechanism involves the TBAF-promoted elimination of HBr from *o*-substituted *gem*-dibromoolefins 1b/1c to form intermediate 176, which undergoes intramolecular nucleophilic addition, giving 2-bromobenzofuran 54. Concurrently, sodium arylsulfinate 175 reacts with Pd^II^, forming intermediate 179, which undergoes desulfitation to deliver aryl-Pd^II^-X species 180 and SO_2_. 180 then reacts with 177, generated *via* oxidative addition of 2-bromobenzofuran 54 to Pd^0^, resulting in intermediate 178 through transmetalation. Reductive elimination of 178 affords the final product 59 and regenerates Pd^0^.

**Scheme 44:**
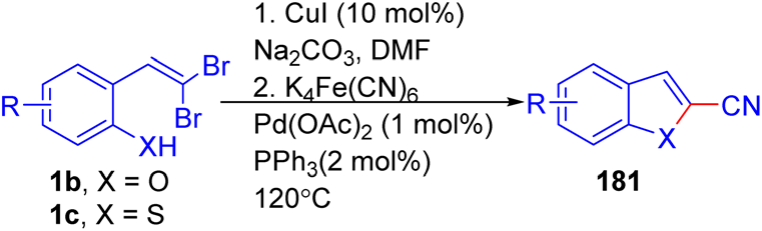
Generation of 2-cyanobenzofurans(thiophenes).

A one-pot palladium and copper-catalyzed Ullmann reaction/cyanation of *o*-substituted *gem*-dibromoolefins 1b/1c*via* K_4_Fe(CN)_6_ as a non-toxic cyanating reagent, to generate 2-cyanobenzofurans(thiophenes) 181 was described by Zhou and coworkers (Scheme 44).^68^

The reaction scope was tested with various *o*-substituted *gem*-dibromoolefins 1b/1c, yielding products 181 with good to excellent yields. The presence of electron-donating or halogen groups at the *para*-position of phenols resulted in higher yields compared to strong electron-withdrawing groups such as NO_2._

**Scheme 45:**
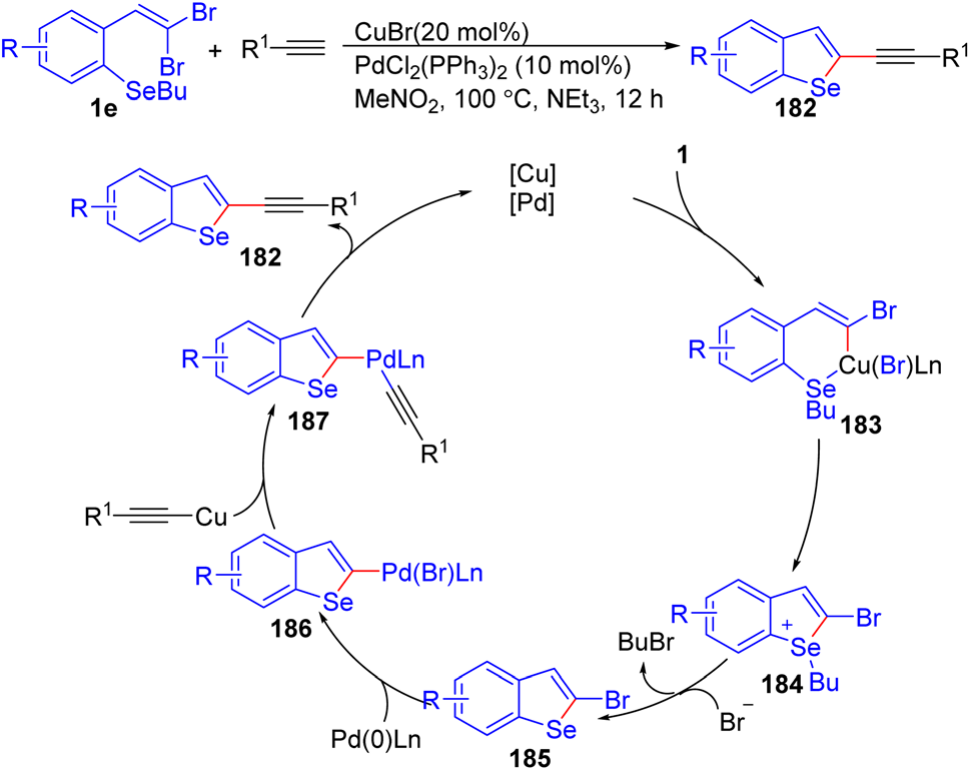
Synthesis of functionalized 2-alkynylbenzo[*b*]selenophenes.

Another excellent example of utilizing *o*-substituted *gem*-dibromoolefins 1e to construct 2-substituted benzo[*b*]selenophenes 182 was reported by Bilheri *et al.* using PdCl_2_/PPh_3_ as the catalyst and CuBr as the cocatalyst, they synthesized a series of functionalized 2-alkynylbenzo[ b]selenophenes 182*via* sequential cyclization/Sonogashira cross-coupling reactions (Scheme 45).^69^ Through several experiments, the authors hypothesized the reaction commences with the oxidative addition of copper to *o*-substituted *gem*-dibromoolefins 1b/1c, leading to 2-bromobenzo[*b*]selenophene 185*via* an intramolecular Ullmann reaction, followed by nucleophilic substitution on the selenium atom. Subsequently, the oxidative addition of 2-bromobenzo[*b*]selenophene 185 to the palladium species leads to formation intermediate 186. In the next step, the copper-activated alkyne reacts with intermediate 184, affording intermediate 187. Finally, reductive elimination of intermediate 187 delivers the desired product 182 while regenerating the catalysts for further cycles. This concise sequence demonstrates the efficiency of the combined copper- and palladium-catalyzed reactions for the synthesis of functionalized benzo[*b*]selenophene derivatives 182.

**Scheme 46:**
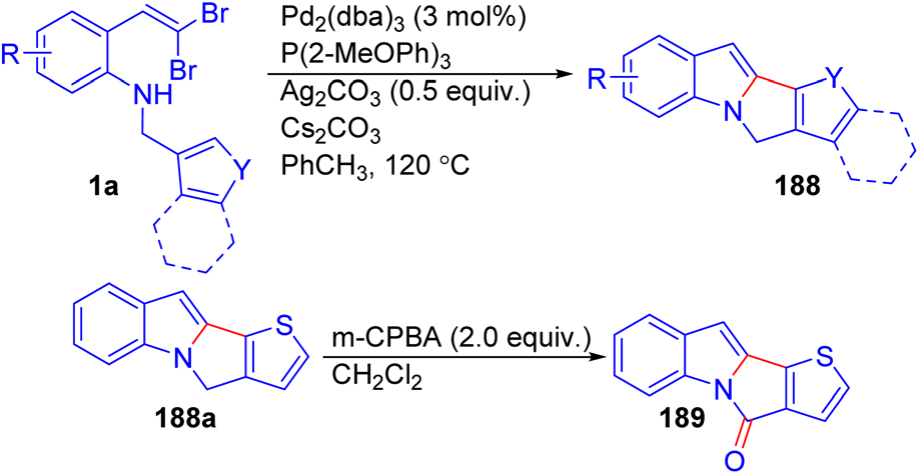
Synthesis of tetracyclic and pentacyclic indole derivatives.

Bryan and Lautens developed a Pd-catalyzed domino Buchwald–Hartwig amination and direct arylation reaction to access tetracyclic and pentacyclic indole derivatives 188 from *o*-substituted *gem*-dibromoolefins 1a (Scheme 46).^70^ During optimization, the authors found that increasing the ligand/Pd ratio improved yields, suggesting that liberated halides were poisoning the catalyst. To resolve this issue, they introduced Ag_2_CO_3_ to sequester the halides, resulting in cleaner reactions and enabling a lower ligand/Pd ratio. The reaction demonstrated broad functional group tolerance, accommodating halides, electron-donating, and electron-withdrawing groups. Notably, the selective oxidation of the methylene group in the desired product using *m*-CPBA delivered lactam 189.

**Scheme 47:**
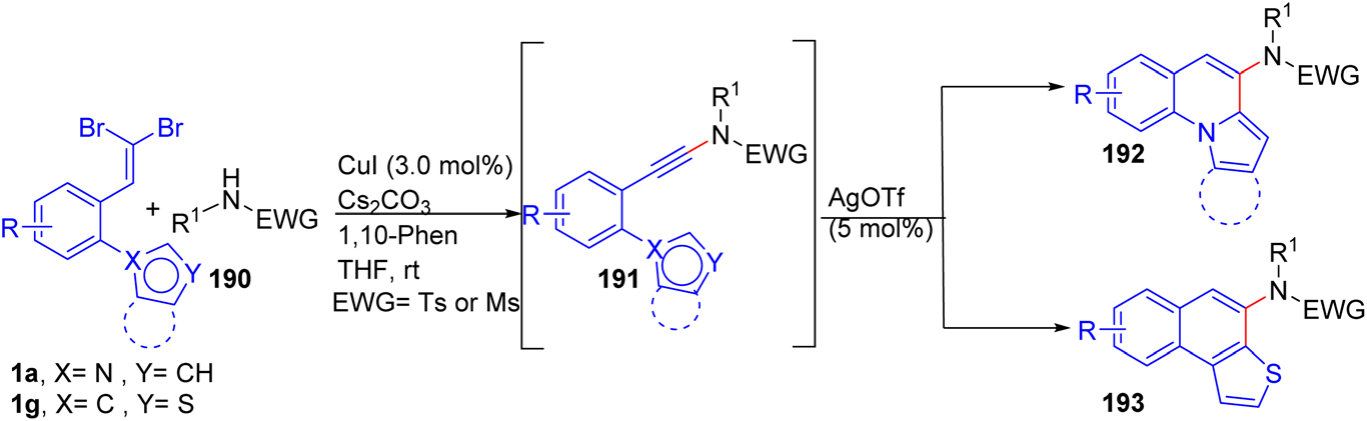
Synthesis of pyrrolo-/indolo[1,2-*a*]quinolines and naphtho[2,1-*b*]thiophenes.

A two-step, one-pot cyclization synthesis of pyrrolo-/indolo[1,2-*a*]quinolines 192 and naphtho[2,1-*b*]thiophenes 193 from *o*-substituted *gem*-dibromoolefins 1 and sulphonamides 190*via* Cu(ii)-catalyzed dehydrobromination and Ag(i)-catalyzed activation of the triple bond under mild conditions was reported by Perumal and co-workers (Scheme 47).^71^ Importantly, the several products exhibited photophysical properties. The formation of ynamide 191 as the key intermediate, which is generated from reaction of *o*-substituted *gem*-dibromoolefins 1 with sulphonamides 190 in the presence of Cu as catalyst in this reaction was suggested by the authors.

**Scheme 48:**
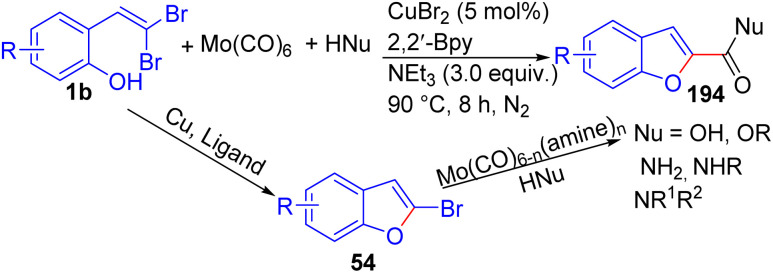
Synthesis of a diverse range of benzofuran-2-carboxylic acids, esters, and amides.

Hu *et al.* have demonstrated a versatile, modular synthesis of a diverse range of benzofuran-2-carboxylic acids, esters, and amides 194, this streamlined approach integrates a Cu-catalyzed intramolecular C–O coupling and a Mo(CO)_6_-mediated intermolecular carbonylation reaction in a single-step procedure, efficiently furnishing the desired products 194 in high yields without the need for Pd catalysts or CO gas (Scheme 48).^72^ The optimal reaction conditions involved were 5 mol% CuBr_2_ as the catalyst, 5 mol% 2,2′-Bpy as the ligand, 3.0 equiv. of Et_3_N as the base, 0.6 equiv. of Mo(CO)_6_ as the solid CO source, in ethanol, at 90 °C for 8 hours under nitrogen atmosphere. In general, a wide range of functional groups such as Me, OMe, *t*-Bu, Ph, F, Cl, Br, COOCH_3_, and other similar substituents were well-tolerated in the reaction for the synthesis of benzofuran-2-carboxylic esters, acids, and amides 194. However, the 4-nitro substituent was not compatible with this reaction, giving complex reductive byproducts, and ammonia as an external *N*-nucleophile was less active than organic amines, leading to a lower yield of the desired product. The tandem carbonylation reaction was proposed to follow a two-step mechanism, with a copper-catalyzed intramolecular Ullman cross-coupling of *o-gem*-dibromovinylphenols 1b to form 2-bromobenzofuran intermediates 54, followed by a carbonylative transformation by activated Mo(CO)_6_ in the presence of amine ligands. It should be noted in this reaction, Mo(CO)_6_ acted as both a carbonyl donor and catalyst.

**Scheme 49:**
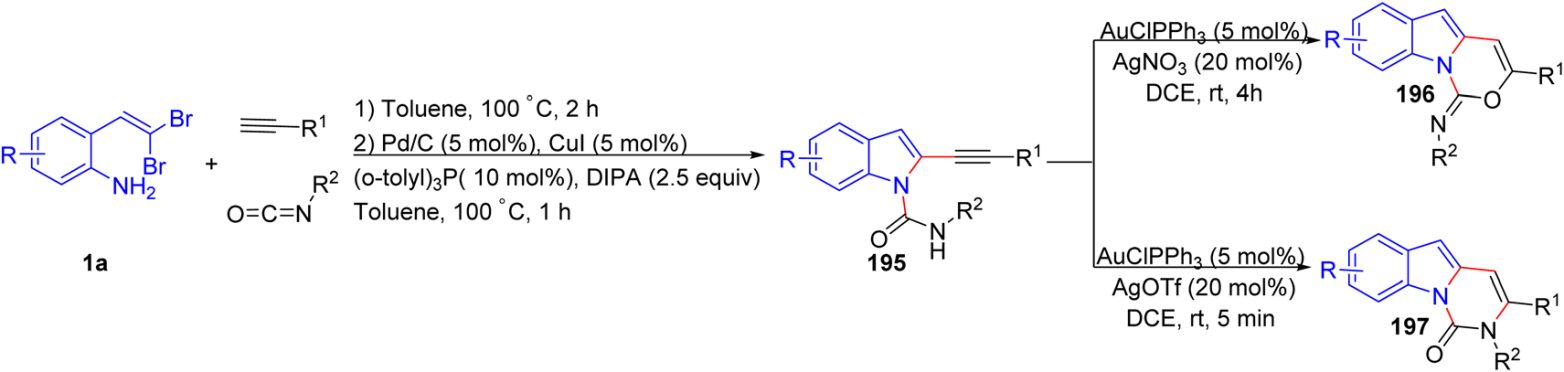
Annulation of o-*gem*-dibromovinyls, isocyanates, and terminal alkynes.

Gupta *et al.* have described annulation of *o*-substituted *gem*-dibromoolefins 1a, isocyanate, and terminal alkyne in the presence of Cu/Pd as catalyst to access an N-1 and C-2 functionalized indoles 195 (Scheme 49).^73^ These products 195 undergo selective 6-*endo* cyclization, yielding either the *O*-cyclized products 196 in the presence of Au(i)/AgNO_3_ or the *N*-cyclized products 197 with Au(i)/AgOTf during post-multicomponent reaction modification.

### 2.5. Indium-catalyzed cyclization

Indium has emerged as an important transition metal in catalytic reactions due to its inert character, high electronegativity, ease of handling, stability in air and moisture, and greater resistance to oxidation compared to other metals. Moreover, its recyclability and high tolerance toward a wide range of chemical substrates and functional groups have led to its increasing use in organic reactions in recent years.^74^

**Scheme 50:**
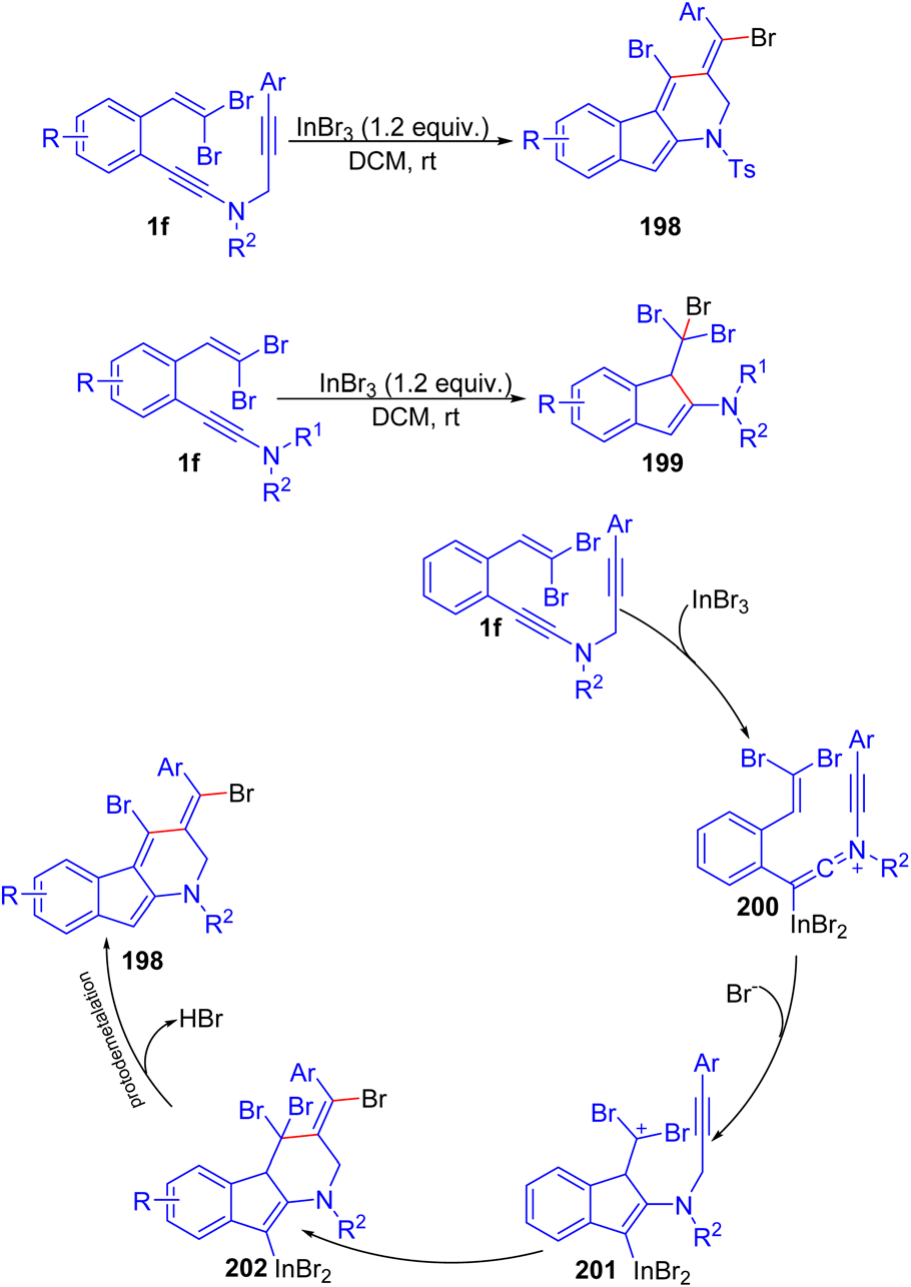
Synthesis of dibrominated 2,3-dihydro-1*H*-indeno[2,1-*b*]pyridine derivatives.

In 2015, Weng *et al.* reported synthesis of dibrominated 2,3-dihydro-1*H*-indeno[2,1-*b*]pyridine derivatives 198*via* reaction of *o*-substituted *gem*-dibromoolefins 1f in the presence of InBr_3_ at 80 °C under nitrogen for 10 minutes (Scheme 50).^75^

These products 198 show potential in medicinal chemistry, as indeno[2,1-*b*]pyridine-based compounds exhibit pharmacological activities. A series of 1 underwent tandem cyclization and desired products 198 were yielded with moderate to high yields for electron-neutral and electron-deficient substrates, while electron-donating substituents resulted in lower yields. Halogen-substituted substrates were successful, but *n*-butyl substituent formed an unidentifiable mixture. A mechanism was suggested that after the formation of the keteniminium cation 200 from ynamide metalation, 200 undergoes 5-*exo-dig* cyclization which leads to formation of cation 201. An anti-addition of bromide and a carbonium ion across the alkyne generates tribrominated compound 202. Subsequent dehydrobromination and protodemetalation result in the formation of 198. In addition, when they performed this reaction at room temperature under nitrogen for 10 minutes, it led to synthesis of 1-tribromomethyl-2-amino-1*H*-indenes 199.

## 3. Metal-free cyclization

While metal-catalyzed transformations have significantly advanced the utility of *ortho*-substituted *gem*-dibromoolefins, metal-free approaches have also emerged as appealing alternatives due to their inherent environmental benefits and cost-effectiveness.^76^ This section discusses recent developments in metal-free cyclization involving *o*-substituted *gem*-dibromoolefins, highlighting their mechanistic pathways, substrate versatility, and synthetic applications.

**Scheme 51:**
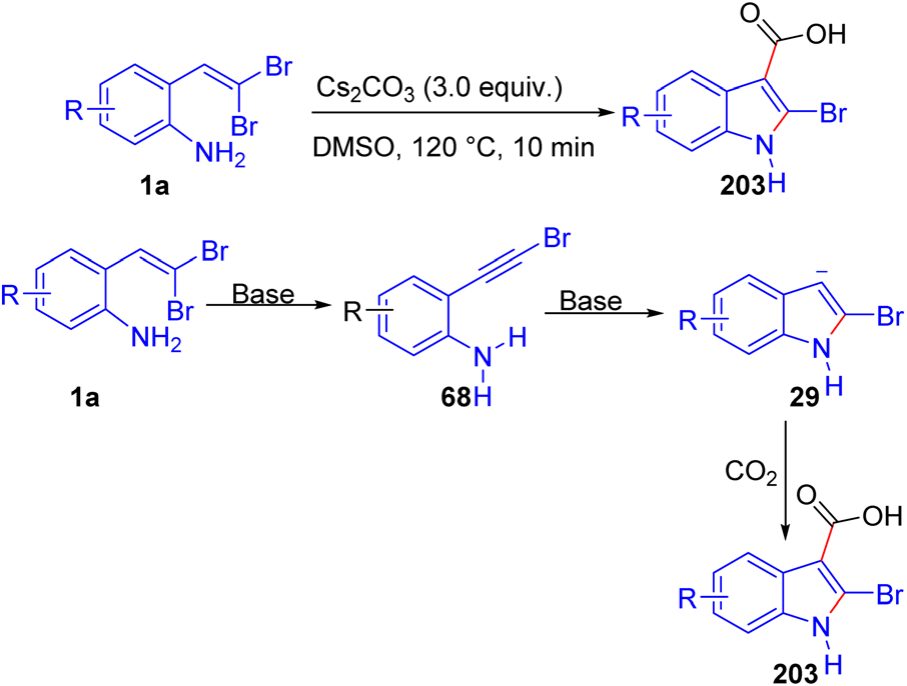
Synthesis of 2-halo-3-carboxyindoles.

Kunzer and Wendt introduced a rapid and efficient method for synthesizing 2-halo-3-carboxyindoles 203 from *o*-substituted *gem*-dibromoolefins 1a using Cs_2_CO_3_ in DMSO under catalyst-free conditions (Scheme 51).^77^ The reaction proceeds optimally at 120 °C, with the formation of an alkynyl bromide intermediate 68, which undergoes cyclization and CO_2_ trapping in a one-pot process, producing the desired indoles 203 cleanly and in high yield after a simple acidic work-up. Electron-donating groups, such as methoxy and methyl substituents, as well as electron-withdrawing groups like nitro and ester functionalities on the aromatic ring, were all well-tolerated, yielding the desired 2-halo-3-carboxyindoles 203 in high purity and yields ranging from moderate to excellent. This wide functional compatibility underscores the method's utility for diverse indole derivatives, including those with sensitive substituents, as the reaction conditions avoid harsh oxidants or transition metals that might otherwise compromise product integrity.

**Scheme 52:**
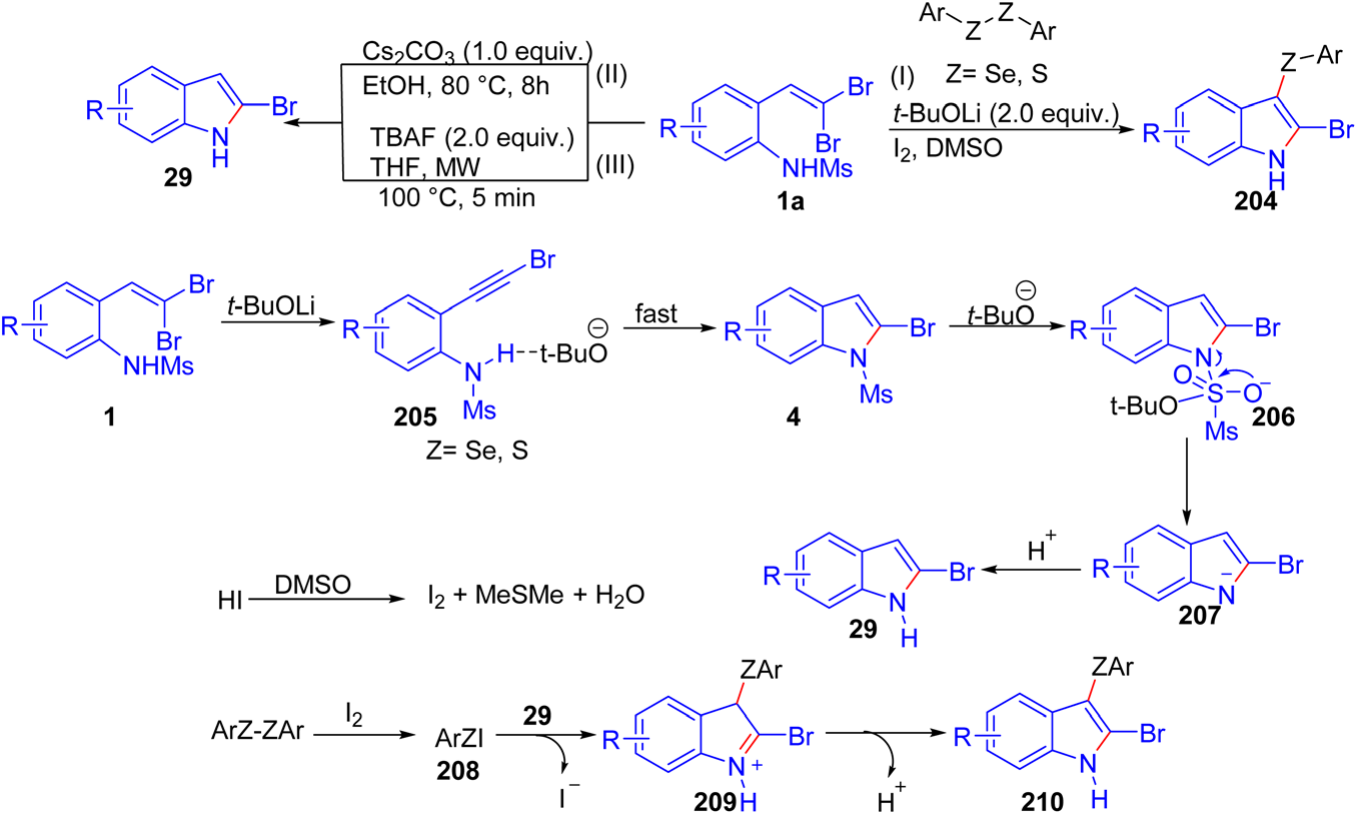
Synthesis of 2-bromo-3-selenyl(sulfenyl)indoles.

Wang *et al.* developed a novel one-pot method for synthesizing 2-bromo-3-selenyl(sulfenyl)indoles 204 by employing *o*-substituted *gem*-dibromoolefins 1 in tandem reactions with diselenides and disulfides (Scheme 52(I)).^78^ Conducted under transition-metal-free conditions, the reaction utilizes *t*-BuOLi and a catalytic amount of I_2_ in DMSO, achieving high regioselectivity and efficient yields. The reaction begins with *t*-BuOLi facilitating the elimination of HBr, generating a phenylethynyl intermediate 205 that subsequently undergoes nucleophilic cyclization to form the indole core 4. For the 3-selenylation step, I_2_ and diselenide react to produce an electrophilic PhSeI species 208, which then undergoes electrophilic addition to the indole 29 at the 3-position, completing the transformation in a sequential manner. Additionally, they demonstrated that this synthetic route also accommodates 2-(*gem*-dichlorovinyl)anilines, which successfully produce 2-chloro-3-selenyl or 3-sulfenyl derivatives. One year later, Wang and colleagues established a streamlined approach for synthesizing 2-bromoindoles 29 through the intramolecular cyclization of *o*-substituted *gem*-dibromoolefins 1a in the presence of Cs_2_CO_3_ as base (Scheme 52(II)).^79^ This metal-free, environmentally considerate method, carried out in ethanol enables the controlled synthesis of either 2-bromoindoles 4 or their *N*-methylsulfonyl-protected counterparts by adjusting Cs_2_CO_3_ stoichiometry. In addition, an excellent illustration of TBAF's utility in metal-free cyclization reactions of dibromoolefins 1a is presented in a method developed by the same group,^80^ where they employ TBAF to efficiently synthesize 2-bromoindoles 29 under microwave-assisted conditions. The reaction proceeds optimally in THF at 100 °C within five minutes, leveraging TBAF's dual role as both base and fluoride source for the cyclization process (Scheme 52(III)).

**Scheme 53:**
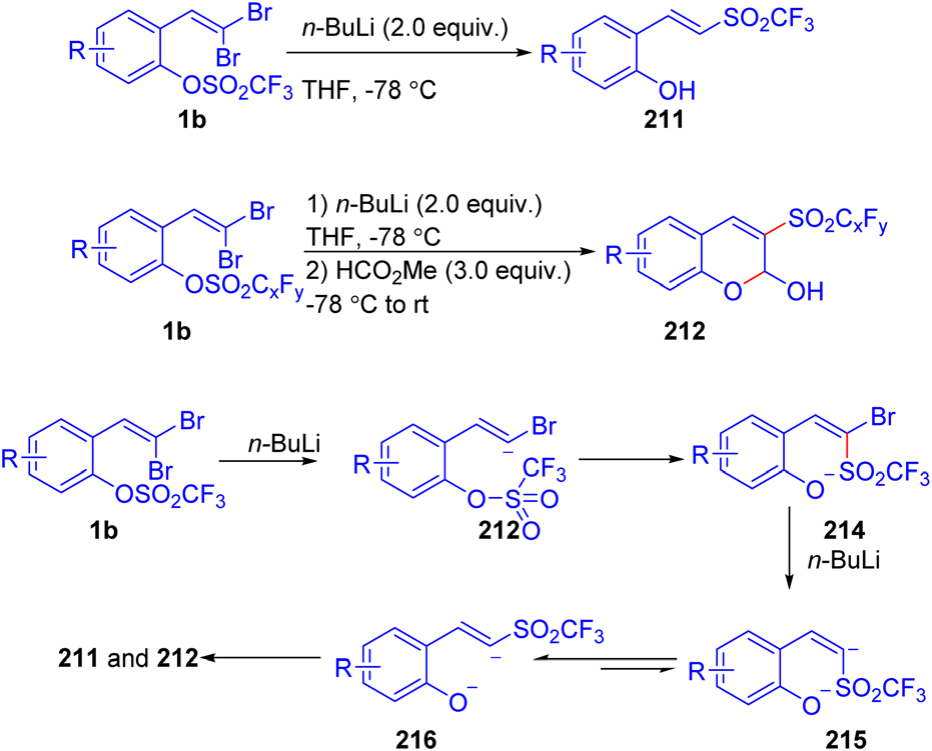
Synthesis of vinyl and heteroaryl triflones.

In a 2013 study, Shibata and colleagues presented a stereoselective synthesis of vinyl and heteroaryl triflones 211/212*via* anionic O → C and N → C trifluoromethanesulfonyl migration reactions (Scheme 53).^81^ These transformations enabled the efficient preparation of vinyl triflones and heteroaryl triflones under mild reaction conditions. A plausible reaction mechanism was provided by authors, as shown in Scheme 53.

**Scheme 54:**
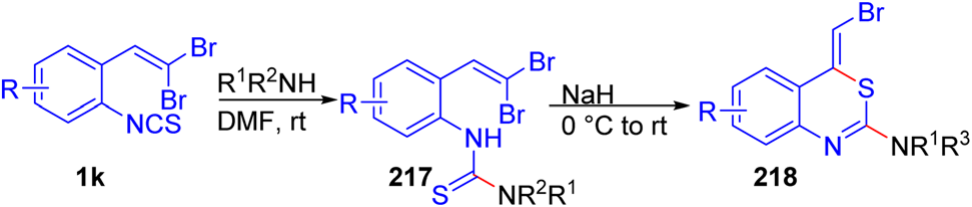
Synthesis for (*Z*)-4-(halomethylidene)-4*H*-3,1-benzothiazin-2-amines.

Kobayashi *et al.* developed a one-pot synthesis for (*Z*)-4-(halomethylidene)-4*H*-3,1-benzothiazin-2-amines 218 using *o*-substituted *gem*-dibromoolefins 1k and secondary amines (Scheme 54).^74^ Mechanistic insights suggest that deprotonation of the thiourea intermediate 217 with NaH produces an amide anion, which undergoes cyclization *via* nucleophilic attack on the β-position of the dihalovinyl group, followed by H-transfer and halide elimination to yield the benzothiazine rings 218. The product 218 configuration was confirmed through NOE experiments, which supported the selective formation of the (*Z*)-isomer. Investigations demonstrated that this sequence reliably yields the desired products 218 with high regioselectivity and in generally good yields across a range of secondary amines, although primary amines failed under the reaction conditions.

**Scheme 55:**
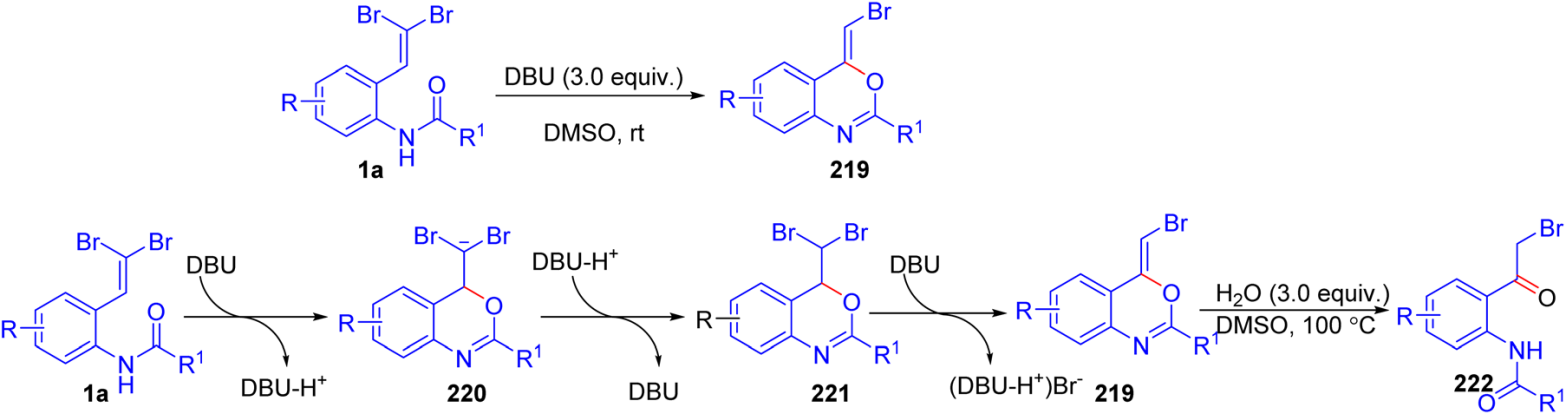
Regio- and stereoselective synthesis of 1,3-benzoxazines.

Garkhedkar *et al.* reported a DBU-promoted regio- and stereoselective synthesis of 1,3-benzoxazines 219 from *o*-substituted *gem*-dibromoolefins 1a, offering a mild, metal-free alternative for constructing these biologically relevant heterocycles (Scheme 55).^82^ Furthermore, the authors extended their method to generate α-bromomethyl ketones 222 through a ring-opening sequence using water in DMSO. Based on several control experiments, it was proposed that the reaction begins with DBU-mediated deprotonation of the *o*-substituted *gem*-dibromoolefins 1a, followed by 6-*exo-trig* cyclization to deliver intermediate 220. Subsequent protonation of intermediate 220 generates a stabilized intermediate 221, which undergoes dehydrohalogenation in the presence of DBU to yield the final 1,3-benzoxazine product 219. Moreover, this reaction produces benzoxazines 219 with a *Z*-configuration, confirmed by 2D-NMR techniques such as NOESY and HMBC. In the subsequent ring-opening step to synthesize *o*-amido phenacyl bromides 222, water acts as the nucleophile, attacking the benzoxazine framework. This results in the cleavage of the benzoxazine ring, forming the phenacyl bromide derivatives 222.

**Scheme 56:**
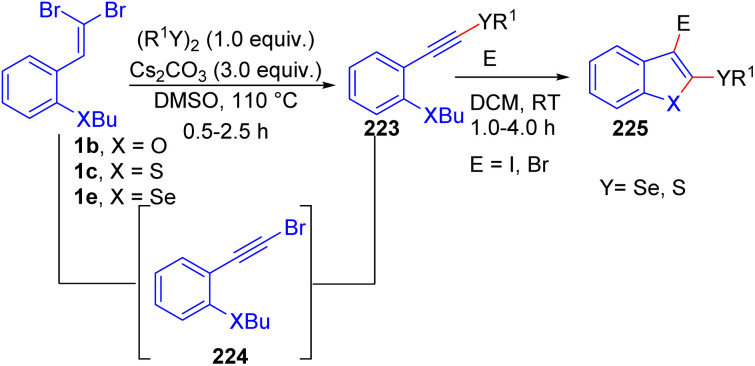
Synthesis of 3-halo-2-organochalcogenylbenzo[*b*]chalcogenophenes.

Rampon and co-workers developed a transition-metal-free one-pot synthesis for 3-halo-2-organochalcogenylbenzo[*b*]chalcogenophenes 225, utilizing reaction between *o*-substituted *gem*-dibromoolefins 1b/1c/1e and diorganoyl dichalcogenides (Scheme 56).^83^ This method efficiently generates 2,3-disubstituted benzo[*b*]chalcogenophenes 225 with yields ranging from moderate to excellent, aiming to streamline synthetic routes by eliminating separate purification steps typically needed for chalcogenoacetylenes. Initial optimization showed that the use of Cs_2_CO_3_ in DMSO afforded the 1-bromoalkyne 224 as target intermediate in high efficiency, a finding corroborated by systematic testing with other bases and solvents. The optimized reaction, conducted at 110 °C, consistently produced high yields within a short time. Mechanistically, the authors propose that base-promoted elimination of *o*-substituted *gem*-dibromoolefins 1 generates a 1-bromoalkyne 224, which further reacts to deliver the chalcogenoacetylene intermediate 223. This intermediate 223 undergoes a nucleophilic cyclization upon the addition of iodine, leading to the desired product 225 in high yield. The method demonstrated broad substrate compatibility, accommodating a range of electron-donating and withdrawing groups without significant impact on yield, especially for *S*-aryl and Se-aryl substituted compounds. The one-pot synthesis was extended using *N*-bromosuccinimide (NBS) as the electrophile, successfully producing desired products 225.

**Scheme 57:**
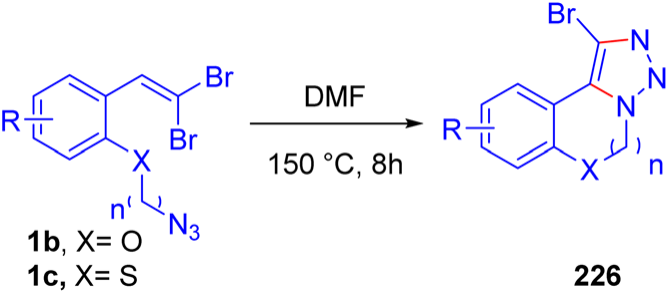
Synthesis of halogen-substituted 1,2,3-triazoles.

Kobayashi *et al.* present a novel synthetic route for halogen-substituted 1,2,3-triazoles 226*via* an intramolecular Huisgen cycloaddition of *ortho*-substituted *gem*-dibromoolefins 1 (Scheme 57).^84^ This method efficiently forms triazole-fused tricyclic benzocompounds 226, offering a straightforward pathway to functional triazole derivatives, which serve as intermediates in various biologically active compounds. The researchers observed that heating azide precursors in DMF at 150 °C gives the desired bromo-substituted triazoles 226 in high efficiency, with the reaction benefiting from a metal-free environment under optimized conditions. To explore the reaction scope, the authors tested a range of halogen substitutions, including bromo, iodo, and fluoro groups, confirming that various halogens are well tolerated. They also demonstrated the versatility of the method by synthesizing sulfur/oxygen-containing triazoles and extending the reaction to triazoles with both 5- and 7-membered rings. These variations yielded products 226 in moderate to excellent yields.

**Scheme 58:**
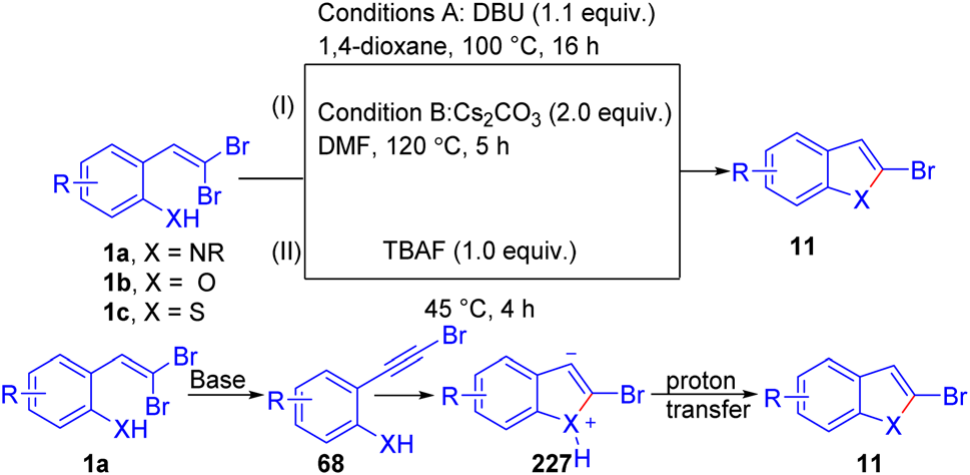
Efficient synthesis of 2-bromobenzoheterocycles.

Lautens *et al.* recently proposed a based-mediated and metal-free method for the efficient synthesis of 2-bromobenzoheterocycles 96 from *o*-substituted *gem*-dibromoolefins 1a/1b/1c, offering a straightforward and practical route to construct these valuable intermediates in pharmaceutical and material chemistry (Scheme 58(I)).^85^ The reaction begins with the base-promoted dehydrohalogenation of the *o*-substituted *gem*-dibromoolefins 1a/1b/1c, leading to the formation of a 1-bromoalkyne intermediate 68. This intermediate 68 undergoes 5-*endo-dig* cyclization, forming the core benzoheterocycle structure 11. The use of strong bases, such as DBU, was found to be crucial in facilitating the dehydrohalogenation step, allowing the formation of the 1-bromoalkyne intermediate 68, which then cyclizes under mild conditions. The authors confirmed their hypothesis through several mechanistic studies, which included deuterium-labeling experiments, confirming the presence of a proton exchange at the C3 position of the final benzoheterocycle product. Additionally, the authors conducted a series of control experiments to demonstrate the necessity of both the base and the bromo group in promoting cyclization. These studies confirmed that the 1-bromoalkyne intermediate 68 plays a key role in driving the reaction towards the benzoheterocycle product 11, highlighting the importance of base-mediated cyclization in this transformation. It should be noted, Chen and associates in 2011, reported a TBAF-promoted intramolecular cyclization of *gem*-dibromoolefins to efficiently synthesize 2-bromobenzofurans and 2-bromobenzothiophenes 11 under metal-free conditions (Scheme 58(II)).^86^ This methodology utilizes readily available *o*-substituted *gem*-dibromoolefins 1a/1b/1c and addresses challenges associated with traditional halogenation methods, which often require toxic electrophilic halogen sources and yield a mixture of regioisomers. Exploring the reaction scope, they demonstrated that various electron-donating and electron-withdrawing substituents on the benzene ring were well-tolerated, yielding the desired products 11 in high efficiency.

## 4. Conclusions

This review comprehensively summarizes the advancements made, in a categorical manner, in the field of cyclic compound formation through cyclization reactions of *o*-substituted *gem*-dibromoolefins involving various mechanistic pathways. Two types of cyclization reactions are summarized, based on whether the reactions occur under transition-metal catalysis or under metal-free conditions promoted by a base. The reactions presented and discussed illustrate the versatility of these building blocks for the synthesis of a wide range of carbo- and heterocyclic ring systems using *o*-substituted *gem*-dibromoolefins. As shown in this review, Pd, Cu, Rh, and In have acted as practical and efficient metal catalysts in transformations to access cyclic compounds. Furthermore, the application of other metal catalysts has been less investigated in the field of *o*-substituted *gem*-dibromoolefin chemistry, and it will be an intriguing opportunity to explore their catalytic activity. On the other hand, some transition-metal-free cyclization reactions of *o*-substituted *gem*-dibromoolefins can be employed for the preparation of cyclic compounds with very high efficiency. The environmentally benign nature of the method plays a crucial role in the selection of the approach. While significant progress has been made, key challenges remain in utilizing *o*-substituted *gem*-dibromoolefins, particularly in achieving higher selectivity, milder reaction conditions, and improved scalability. Nevertheless, these challenges provide substantial opportunities for further innovation. We hope that this review will encourage organic chemists, especially those interested in *o*-substituted *gem*-dibromoolefin chemistry, to investigate new reactions in this area.

## Conflicts of interest

There are no conflicts to declare.

## Data Availability

This study is a review article, and no new data were generated or analyzed during the course of this research. All data discussed and referenced are available in the publicly accessible sources cited within the article.
